# The homeobox gene *TGIF1* is required for chicken ovarian cortical development and generation of the juxtacortical medulla

**DOI:** 10.1242/dev.199646

**Published:** 2021-08-23

**Authors:** Martin Andres Estermann, Claire Elizabeth Hirst, Andrew Thomas Major, Craig Allen Smith

**Affiliations:** Department of Anatomy and Developmental Biology, Monash Biomedicine Discovery Institute, Monash University, Clayton VIC 3800, Australia

**Keywords:** TGIF1, Ovarian cortex, Juxtacortical medulla, Gonadal development, Sex determination, Chicken embryo

## Abstract

During early embryogenesis in amniotic vertebrates, the gonads differentiate into either ovaries or testes. The first cell lineage to differentiate gives rise to the supporting cells: Sertoli cells in males and pre-granulosa cells in females. These key cell types direct the differentiation of the other cell types in the gonad, including steroidogenic cells. The gonadal surface epithelium and the interstitial cell populations are less well studied, and little is known about their sexual differentiation programs. Here, we show the requirement of the homeobox transcription factor gene *TGIF1* for ovarian development in the chicken embryo. *TGIF1* is expressed in the two principal ovarian somatic cell populations: the cortex and the pre-granulosa cells of the medulla. *TGIF1* expression is associated with an ovarian phenotype in estrogen-mediated sex reversal experiments. Targeted misexpression and gene knockdown indicate that TGIF1 is required, but not sufficient, for proper ovarian cortex formation. In addition, *TGIF1* is identified as the first known regulator of juxtacortical medulla development. These findings provide new insights into chicken ovarian differentiation and development, specifically cortical and juxtacortical medulla formation.

## Abstract (precis)

**Summary:** The homeobox transcription factor TGIF1 is required for proper ovarian sex differentiation in chicken embryos, regulating development of the cortical and juxtacortical medulla, independently of the supporting cell sex lineage.

## INTRODUCTION

Vertebrate gonadal sex differentiation is a unique process whereby the embryonic gonadal primordium typically adopts either an ovarian or a testicular fate (Brennan and Capel, 2004; Stévant and Nef, 2019; Rotgers et al., 2018). This process involves sexually dimorphic gene expression that activates one pathway and represses the other, making testis and ovary formation mutually exclusive (Kim et al., 2006; Li et al., 2017). The undifferentiated gonad initially comprises the same set of uncommitted cell lineage precursors: so-called supporting cells, steroidogenic progenitors, germ cells and some other less well-defined cells (Lin et al., 2017; Lin and Capel, 2015; Nef et al., 2019). The first cell lineage to differentiate is the supporting cell lineage, giving rise to Sertoli cells in the male gonad and pre-granulosa cells in females (Niu and Spradling, 2020; Chen et al., 2017; Zhang et al., 2015a). These cells are then thought to direct other lineages down the testicular or ovarian pathways, respectively (Lin and Capel, 2015; Rotgers et al., 2018; Wear et al., 2017; Gustin et al., 2016). In males, Sertoli cells organize into testis cords and signal to neighboring steroidogenic precursors to become sex steroid hormone-producing fetal Leydig cells in the developing testis (Yao et al., 2002). The same lineage gives rise to thecal cells in the developing ovary, although this requires interactions with the germ cells (Liu et al., 2015; Stévant et al., 2019). Germ cells themselves follow a fate governed by signals from the somatic component of the gonad, giving rise to spermatogonia in the testis and oogonia in the ovary (Barrios et al., 2010; Bowles et al., 2010; DiNapoli et al., 2006; Spiller et al., 2017). Other cell types in the embryonic gonad are less well characterized, including the gonadal surface epithelium (the source of the supporting and some of the steroidogenic cell lineages in mouse) and non-steroidogenic ‘interstitial’ cells derived from the surface epithelium or the adjacent mesonephric kidney (DeFalco et al., 2011; Rotgers et al., 2018; Svingen and Koopman, 2013; Stévant and Nef, 2019).

Gonadal sex differentiation has been widely studied as a paradigm for the molecular genetic regulation of development. In the mouse model, the Y chromosome-linked *Sry* gene initiates the testis developmental program (Koopman et al., 1991; Sinclair et al., 1990; Hacker et al., 1995; Kashimada and Koopman, 2010). It activates the related *Sox9* gene, leading to Sertoli cell differentiation, and subsequent downstream signaling to channel other cell types down the male pathway (Sekido et al., 2004; Sekido and Lovell-Badge, 2008; Qin and Bishop, 2005; Li et al., 2014; Gonen et al., 2017). In female mammals (genetically XX), the absence of *Sry* allows activation of the signaling molecule R-spondin 1, activation of Wnt4 and stabilization of β-catenin, and downstream expression of the transcription factor Foxl2 (Li et al., 2017; Parma et al., 2006; Tomizuka et al., 2008; Maatouk et al., 2008; Chassot et al., 2008; Jordan et al., 2003). The molecular regulation of gonadal sex differentiation is still incompletely understood, specifically with regard to cell types other than the key supporting cell lineage. Recently, bulk and single-cell RNA sequencing (RNA-seq) approaches have expanded the list of genes implicated in gonadal sex differentiation (Stévant et al., 2019, 2018; Estermann et al., 2020). Many genes uncovered by these approaches remain to be functionally analyzed.

Our understanding of vertebrate gonadal development has been enhanced through comparative studies in non-mammalian models. Although several core genes required for gonadal sex differentiation are conserved across species (Sox9 in the testis and Foxl2 in the ovary, for example) (Kent et al., 1996; Major et al., 2019; Capel, 2017), upstream master sex genes can be divergent. Sry is absent in non-mammals, and so other molecular sex triggers must exist. Among egg-laying vertebrates, the transcription factor DMRT1 plays a major role, analogous to Sry. DMRT1 acts as a master sex switch in birds and in many reptiles with temperature-dependent sex determination, inducing testis development (Smith et al., 2009; Sun et al., 2017; Lambeth et al., 2014; Shioda et al., 2021; Ioannidis et al., 2021). The chicken embryo, in particular, has provided valuable insights into the genetic regulation of gonadal sex differentiation, the evolution of genetic sex switches, and the cell biology of gonadogenesis (Sekido and Lovell-Badge, 2007; Guioli et al., 2020; Smith and Sinclair, 2004; Estermann et al., 2020). As embryonic development occurs *in ovo* and is accessible for experimental manipulation, the chicken provides a powerful model for functional analysis of gonadal sex-determining genes (Schmid et al., 2015). This model has been particularly useful for elucidating the cellular events underpinning gonad formation. Chickens have a ZZ male/ZW female sex chromosome system, in which the Z-linked *DMRT1* gene operates as a master testis regulator via a dosage mechanism (two doses in males) (Ioannidis et al., 2021). In ZZ embryos, the gonads differentiate into bilateral testes. As in mammals, the seminiferous cords form in the inner gonadal medulla in chicken, comprising Sertoli cells that enclose germ cells (Smith and Sinclair, 2004). The male germ cells undergo mitotic arrest, entering meiosis only after hatching (Ayers et al., 2013). In the female chicken gonad, the inner medulla is the site of aromatase gene expression. Aromatase catalyzes the synthesis of estrogens, which are essential for ovarian differentiation in birds (and other egg-laying vertebrates) (Scheib, 1983; Vaillant et al., 2001b; Pieau and Dorizzi, 2004).

The avian model is particularly useful for shedding light on the role of the gonadal surface epithelium. In mouse, the surface epithelium gives rise to the supporting cell lineage and then contributes to the steroidogenic lineage (Lin et al., 2017; Stévant et al., 2018, 2019). In chicken, the surface epithelium gives rise to non-steroidogenic interstitial cells, not the supporting cell lineage as in mouse (Estermann et al., 2020; Sekido and Lovell-Badge, 2007). Prior to gonadal sex differentiation in chicken, the gonadal epithelial layer is thicker in the left gonad than the right gonad (in both sexes) (Omotehara et al., 2017; Guioli et al., 2014). During sex differentiation, this asymmetry becomes less marked in males (Guioli et al., 2014). However, asymmetry is maintained and becomes very pronounced in females (Smith and Sinclair, 2004). The right gonad regresses in female birds, whereas the epithelium of the left gonad continues to proliferate to become a thickened cortex (Guioli et al., 2014). Increased proliferation in the left cortex, rather than increased apoptosis in the right cortex, is primarily responsible for the observed asymmetric cortical development and is driven by estrogen (Ishimaru et al., 2008; Guioli et al., 2020). The left cortex is crucial for ovarian development in the avian model. It contains both somatic cells and proliferating germ cells that enter meiosis to later arrest at prophase I around mid-development (Ukeshima, 1996; Smith et al., 2008). Immediately beneath the cortex of the left ovary, interstitial medullary cells form a compact region called the juxtacortical medulla (JCM). We have previously shown through single-cell RNA-seq that the cells of the JCM are non-steroidogenic and derive from the ovarian surface epithelium (Estermann et al., 2020). The functional significance of the JCM is unclear, although at later stages it expresses enzymes involved in retinoic metabolism, and retinoic acid is implicated in cortical germ cell meiosis (Smith et al., 2008).

We previously conducted bulk RNA-seq to identify genes involved in development of the chicken ovary (Ayers et al., 2015). This screen identified *TGIF1* (TGF-β Induced Factor Homeobox 1). *TGIF1* encodes a homeobox transcription factor that belongs to the superfamily of TALE homeodomain proteins known to control many developmental processes, including gastrulation, cell proliferation, and differentiation (Wotton et al., 1999a,b; Lorda-Diez et al., 2009; Melhuish and Wotton, 2000; Wotton et al., 2001; Liu et al., 2014; Powers et al., 2010). It has not previously been associated with gonadal sex differentiation in any species. In the current study, we describe the role of *TGIF1* in chicken ovarian development. *TGIF1* is specifically upregulated in female gonads at the onset of sexual differentiation, is expressed in cortical and pre-granulosa cells and is associated with the ovarian phenotype. Overexpression and knockdown of *TGIF1* show that it is required for the formation of the female cortex and the JCM. The data suggest that TGIF1 is required for proper ovarian development in the avian model, acting downstream of estrogen signaling.

## RESULTS

### *TGIF1* but not *TGIF2* shows sexually dimorphic expression in embryonic chicken gonads

**Fig. 1.**
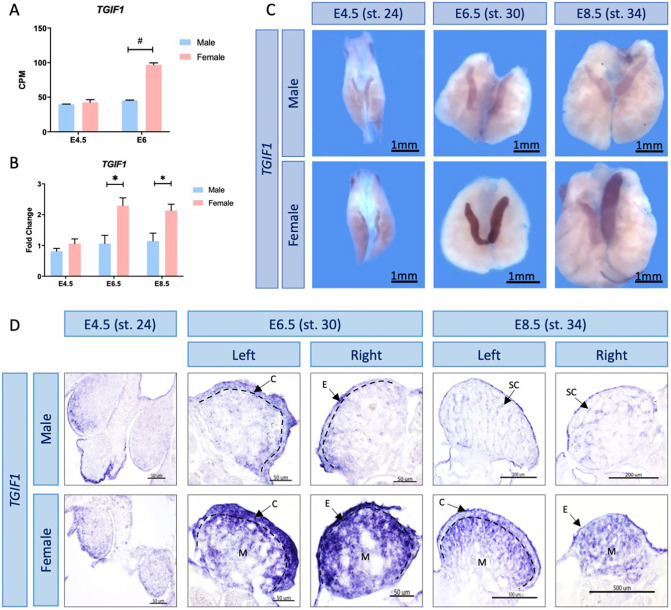
***TGIF1* expression profile in chicken gonads.** (A) *TGIF1* gonadal RNA-seq mRNA expression levels in count per million (CPM) at the blastoderm stage, before (E4.5) and at the onset of (E6) sex determination. #, false discovery rate (FDR) <0.001. Bars represent mean±s.e.m., *n*=2. (B) *TGIF1* gonadal mRNA was quantified by qRT-PCR. Expression level is relative to β-actin and normalized to E4.5 female. Bars represent mean±s.e.m., *n*=6. *adjusted *P*<0.05. Multiple *t*-tests and Holm–Sidak post-hoc test. (C) *TGIF1* time course mRNA expression in embryonic chicken gonads, as assessed by WISH. (D) Sections of the *TGIF1* WISH. Arrows indicate the cortex (C), epithelium (E), medulla (M) and seminiferous cords (SC). Dashed black lines indicate the cortical-medulla limit.

TGIF1 was first identified as a candidate gene in avian gonadal sex differentiation from gonadal RNA-seq performed in our laboratory (Ayers et al., 2015). Differential expression analysis showed that *TGIF1* mRNA expression was significantly higher in female compared with male gonads at the onset of sex differentiation. This corresponds to embryonic day (E) 6 [Hamburger and Hamilton stage (HH) 29; Hamburger and Hamilton, 1951] (Fig. 1A). To validate sexually dimorphic expression, *TGIF1* qRT-PCR was performed on male and female gonads before, during and after gonadal sex differentiation: E4.5 (HH stage 24), E6.5 (HH stage 30) and E8.5 (HH stage 34), respectively. Quantitative RT-PCR showed a significant increase in *TGIF1* expression in female gonads from the onset of sexual differentiation (E6.5-8.5; HH stage 30-34) (Fig. 1B), consistent with the RNA-seq data. *TGIF1* was also expressed in male gonads, but at consistently lower levels. *TGIF1* has a paralog, *TGIF2*, with which it shares spatial and temporal expression in other developmental contexts (Shen and Walsh, 2005). *TGIF2* expression was not sexually dimorphic in the gonad RNA-seq data (Fig. S1A). This was also confirmed by qRT-PCR (Fig. S1B).

### *TGIF1* is expressed in the ovarian cortex and medullary pre-granulosa cells

For spatial expression analysis of *TGIF1*, whole-mount *in situ* hybridization (WISH) was performed on male and female gonads at different developmental time points: before (E4.5/HH 24), during (E6.5/stage 30) and after (E8.5/stage 34) sexual differentiation. *TGIF1* mRNA expression was stronger in female compared with male gonads from E6.5/HH stage 30 (Fig. 1C), consistent with the RNA-seq and qRT-PCR results. In developing ovaries, *TGIF1* mRNA was localized in the outer gonadal cortex and in the underlying medulla region (Fig. 1D). In males, expression was detected at the surface epithelium at E6.5, although this expression was weaker than that in the developing ovary (Fig. 1D). After the onset of sexual differentiation at E8.5 in males, weak expression was detected in the seminiferous cords of the medulla.

**Fig. 2.**
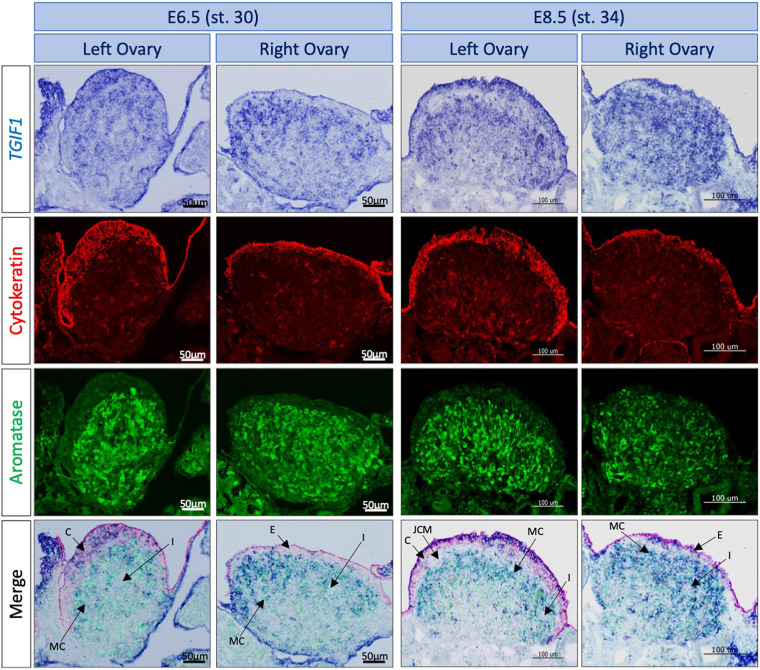
***TGIF1* expression colocalizes with key ovarian cells.***TGIF1* WISH was performed on E6.5 and E8.5 female urogenital tissue. Immunofluorescence staining against cytokeratin (cortical cells marker) and aromatase (pre-granulosa cells marker) was carried out on 10 μm-thick sections. *TGIF1* expression colocalizes with both female markers in left ovaries. Arrows indicate the interstitial (I), cortical (C), medullary cord (MC), epithelial (E) and JCM cells.

The developing chicken ovary comprises two distinct compartments: the outer cortex, which becomes thickened in the left ovary and is the site of oogenesis, and an inner medulla comprising interstitial, supporting and steroidogenic cells (Smith and Sinclair, 2004; Estermann et al., 2020). Colocalization with specific markers was performed to determine the cell types expressing *TGIF1* in the ovary. Aromatase and cytokeratin immunofluorescence were performed following *TGIF1 in situ* hybridization on tissue sections, at E6.5 and E8.5 (Fig. 2). Aromatase marks estrogenic pre-granulosa cells of the medulla, whereas cytokeratin marks cortical cells. In the left ovary, *TGIF1* was expressed in the cortical cells, colocalizing with cytokeratin. Lack of *TGIF1* expression in the right female gonad corresponded with the lack of a proliferating cortex. *TGIF1* mRNA also colocalized with the medullary pre-granulosa marker aromatase in both left and right gonads. *TGIF1* was not expressed in the interstitial cells between medullary cords or in the JCM of female gonads (Fig. 2). In summary, *TGIF1* expression was restricted to cortical and pre-granulosa cells, both key cell types in ovarian development and differentiation.

### *TGIF1* expression is sensitive to estrogens

**Fig. 3.**
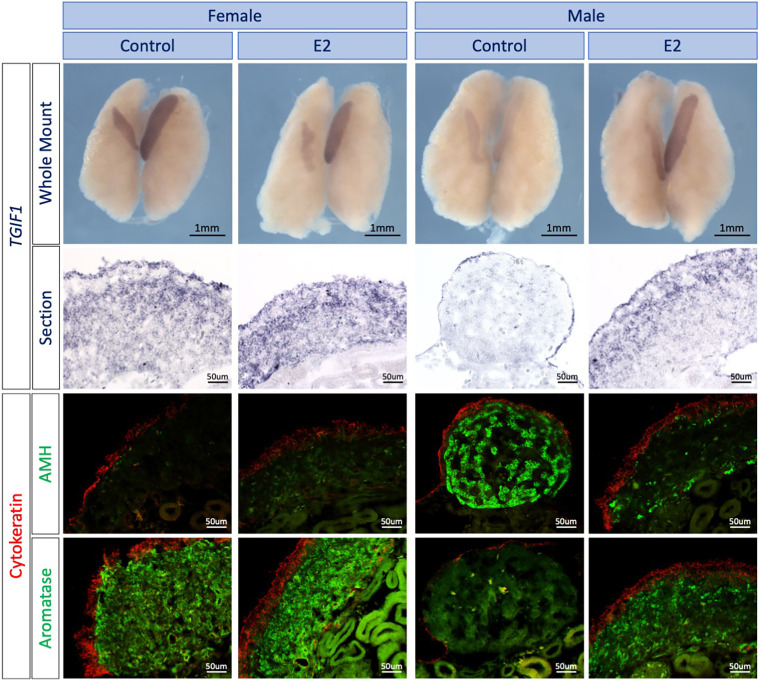
**Estrogens induce *TGIF1* expression in ZZ gonads.***TGIF1* WISH was performed on E9.5 male and female urogenital tissue treated *in ovo* with 17β-estradiol (E2) or vehicle (Control). Tissues were sectioned and immunofluorescence for aromatase (pre-granulosa marker) or AMH (Sertoli cell marker) and cytokeratin (cortical marker) was performed to evaluate the efficacy of the sex reversal.

Ovarian differentiation in birds is regulated by estrogen, catalyzed by the female-restricted enzyme P_450_ aromatase. *In ovo* injection of 17β-estradiol (E2) or the aromatase inhibitor fadrozole cause feminization and masculinization of the gonads, respectively (Bannister et al., 2011; Guioli et al., 2020; Elbrecht and Smith, 1992; Scheib, 1983). In particular, estrogen is required for formation of the left ovarian cortex (Guioli et al., 2020). To determine whether *TGIF1* is responsive to estrogen signaling during ovarian development, sex reversal experiments were conducted. *TGIF1* was assayed following masculinization of female embryos with fadrozole, which inhibits aromatase enzyme activity, or by applying estrogen to male embryos to induce feminization. *TGIF1 in situ* hybridization was performed on E9.5 (stage 37) male and female urogenital systems (UGS) treated with 17-β-estradiol (E2) or vehicle (control) at E3.5 (stage 19) (Fig. 3). Male gonads treated with E2 were morphologically feminized, with female-like asymmetry, characterized by a larger left and smaller right gonad. These gonads also showed structural organization typical of an ovary, with a thickened cortex (cytokeratin positive), aromatase-positive pre-granulosa cells in the medulla and downregulation of the testis marker anti-Müllerian hormone (AMH) (Fig. 3). *TGIF1* expression was upregulated in males treated with E2, compared with the vehicle control, showing a similar expression pattern to females (Fig. 3).

**Fig. 4.**
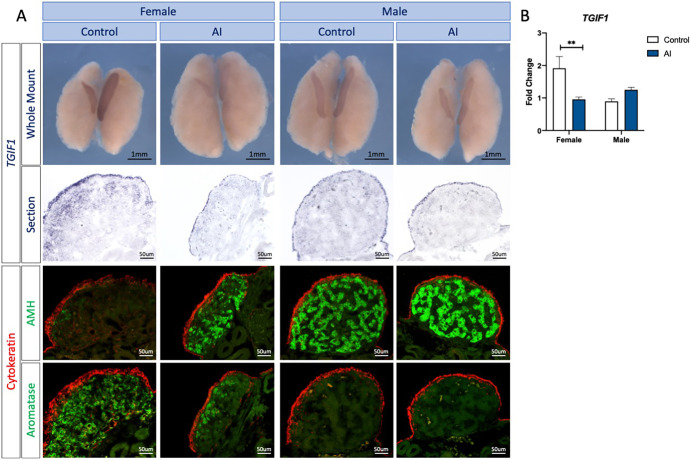
**Estrogen synthesis inhibition by fadrozole results in downregulation of TGIF1 in ZW gonads.** (A) *TGIF1* WISH was performed on E9.5 male and female urogenital tissue treated *in ovo* with the aromatase inhibitor fadrozole (AI) or vehicle (Control). Tissues were sectioned and immunofluorescence for aromatase (pre-granulosa marker) or AMH (Sertoli cell marker) and cytokeratin (cortical marker) was performed to evaluate the efficacy of the sex reversal. (B) *TGIF1* qRT-PCR was performed in gonadal samples of E9.5 embryos treated with the aromatase inhibitor (AI) or vehicle (Control). Expression level is relative to β-actin and normalized to the male control. Bars represent mean±s.e.m., *n*=6. **adjusted *P*<0.01. Multiple *t*-tests and Holm–Sidak post-hoc test.

Female gonads treated with the fadrozole aromatase inhibitor (AI) were masculinized, as expected. Female-type gonadal asymmetry was markedly reduced, and gonads showed testicular-like morphology, containing AMH-positive testicular cords, and a reduced cortex and reduced aromatase-positive cells (Fig. 4A). *TGIF1* expression was also reduced in female gonads treated with aromatase inhibitor, consistent with the gonadal sex reversal (Fig. 4A). To quantify this change, *TGIF1* qRT-PCR was performed in E9.5 male and female gonads exposed to AI or vehicle (control). Consistent with the *in situ* hybridization data, female gonads treated with AI showed a significant reduction of *TGIF1* expression in comparison with the vehicle control (Fig. 4B). Altogether, these results indicate that *TGIF1* mRNA expression responds to estrogen during ovarian differentiation in the chicken embryo.

### *TGIF1* overexpression in left testis results in epithelial thickening and JCM formation

As TGIF1 was upregulated in female gonads, misexpression of the gene in males was examined. To study the effects of *TGIF1* misexpression *in ovo*, electroporation of DNA constructs was used. Gonadal epithelial cells can be specifically targeted by electroporating TOL2 plasmid DNA into the coelomic epithelium at E2.5 (HH stage 14), without affecting the underlying medullary cord cell population (Estermann et al., 2020). This method can provide insight into the role of TGIF1 specifically in the surface epithelium/cortex. The *TGIF1* open reading frame (ORF) was cloned into the *TOL2-CAGGS-GFP* vector, which, in the presence of the transposase, integrates into the genome, stably expressing TGIF1 and GFP in the targeted and daughter cells (Sato et al., 2007). The ability of this construct to overexpress TGIF1 was assayed *in vitro* in the DF1 chicken fibroblastic cell line. Cells transfected with TGIF1-overexpressing plasmid expressed a significantly higher level of *TGIF1* compared with the empty plasmid (GFP control) (Fig. S1C).

**Fig. 5.**
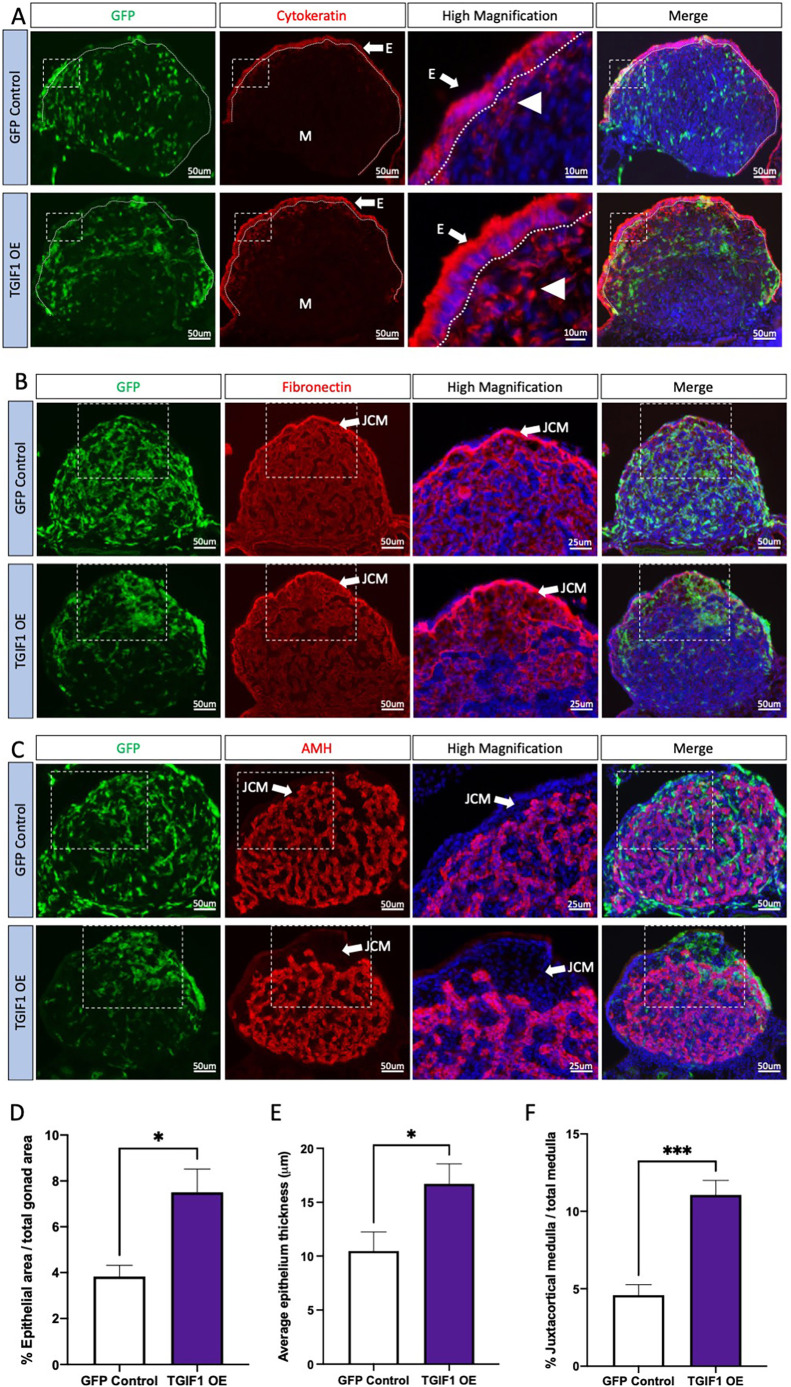
**TGIF1 overexpression in left male gonads results in gonadal feminization.** TOL2-TGIF1 overexpression (TGIF1 OE) or control (GFP Control) plasmids were electroporated in male left E2.5 coelomic epithelium. Gonads were examined at E8.5. (A-C) Immunofluorescence against cytokeratin (epithelial/cortical marker; A), fibronectin (interstitial cell marker; B) and AMH (Sertoli cell marker; C) was performed in transverse sections. Dashed box indicates the magnified area. Dotted line delineates the gonadal epithelium. White arrow indicates the epithelium (E) or JCM, white arrowhead indicates EMT-derived interstitial cell and M indicates medulla. (D) Quantification of the percentage of epithelial area, relative to the total gonadal area in male gonads. (E) Quantification of the average epithelium thickness (in μm) in control or TGIF1-overexpressing male gonads. (F) Quantification of the percentage of JCM area, relative to the total medullar area. Bars represent mean±s.e.m., *n*≥6. Unpaired two-tailed *t*-test. *P<0.05; ***P<0.001.

*TOL2-CAGGS-GFP-T2A-TGIF1* (TOL2-TGIF1-OE) or *TOL2-CAGGS-GFP* (GFP control) plasmids were co-electroporated with a plasmid expressing transposase into the left coelomic epithelium at E2.5 (stage 14). Embryos were collected at E8.5, genetically sexed by PCR, and immunofluorescence was performed for gonadal markers. Fig. 5 shows the results of these experiments in which *TGIF1* was misexpressed specifically in the surface epithelium of male gonads. In the absence of a suitable antibody to detect TGIF1 protein in chicken, GFP was used as a marker of electroporation. As expected, GFP was detected in the gonadal epithelial cells (Fig. 5A), and in the interstitial cells (Fig. 5B) that they generate, but not in supporting cells of males (Fig. 5C) or females (Fig. S2). When *TGIF1* was overexpressed in female gonads, no structural or expression difference compared with the control was found (Fig. S2). In contrast, TGIF1 misexpression in male left gonads resulted in feminization of the surface epithelium. Cytokeratin-positive cells became cuboidal rather than the squamous epithelium that is typical of the testis (Fig. 5A). The surface epithelium of these male gonads became thicker. Image quantification analysis indicated that *TGIF1* overexpression in male gonads resulted in a significant increase of the gonadal epithelial area (Fig. 5D) and the thickness of the epithelium (cell height) (Fig. 5E). In addition, an increase in cytokeratin-positive mesenchymal cells was detected, suggesting augmentation of an epithelial-to-mesenchyme transition (EMT) (Fig. 5A). In male gonads misexpressing *TGIF1*, interstitial cells derived from the coelomic epithelial cells by EMT (GFP^+^, fibronectin^+^) accumulated underneath the epithelial layer, forming dense clusters (Fig. 5B). This accumulation of interstitial cells resembled the organization of the ovarian JCM and resulted in a displacement of the testicular cords (AMH^+^) towards the interior of the gonad (Fig. 5C). Image quantification analysis indicated that TGIF1 misexpression resulted in a significant increase in the JCM area, compared with the controls (Fig. 5F).

To examine its effects on the gonadal medulla, TGIF1-GFP was overexpressed using the RCASBP viral vector. Unlike the TOL2 plasmid used above, this vector can self-propagate and spread to neighboring cells after electroporation. This is important, as it can deliver transgenic expression to the medullary cord population, which cannot be targeted by TOL2 electroporation. The *TGIF1* ORF was cloned into the RCASBP(A)-GFP viral vector. *RCASBP(A)-GFP-T2A-TGIF1* (RCAS-TGIF1-OE) or *RCASBP(A)-GFP* (GFP control) plasmids were electroporated in E2.5 (stage 14) left coelomic epithelium. Urogenital systems were collected at E7.5-8.5, sexed and immunofluorescence was performed for different gonadal markers. Overexpression of *TGIF1* in male gonads using this approach did not alter testicular development. Supporting cells developed normally, and AMH, SOX9 and DMRT1 expression was similar to that of control gonads (Fig. S3A-C). TGIF1 misexpression did not result in downregulation of key Sertoli cell markers, evidenced by the colocalization of TGIF1 (GFP) with DMRT1, SOX9 and AMH (Fig. S3A-C). Additionally, female marker aromatase was not activated (Fig. S4). Instead, the same morphological changes were detected when TGIF1 was overexpressed only in the coelomic epithelial cells via the TOL2 plasmid, i.e. displacement of the supporting cells from the sub-epithelial region (Fig. S3A-C) and an increased thickness of the gonadal surface epithelium, marked by diagnostic cytokeratin staining. The cells of the epithelium adopted a female-like cuboidal morphology instead of the squamous epithelium typical of the testis (Fig. S3D). Altogether, these data suggest that *TGIF1* misexpression does not directly impact Sertoli cell differentiation in chicken gonads.

### *TGIF1* overexpression in right gonads results in epithelial thickening and JCM formation

**Fig. 6.**
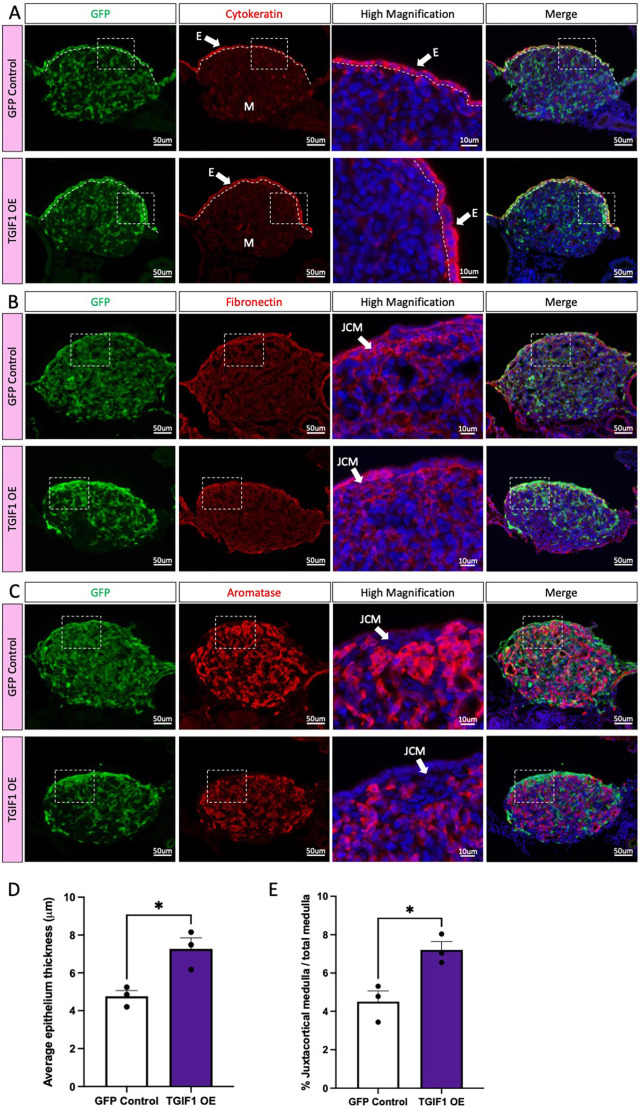
**TGIF1 overexpression in right female gonads results in epithelial thickening and JCM formation.** TOL2 TGIF1 overexpression (TGIF1 OE) or control (GFP Control) plasmids were electroporated in female right E2.5 coelomic epithelium. Gonads were examined at E8.5. (A-C) Immunofluorescence against cytokeratin (epithelial/cortical marker; A), fibronectin (interstitial cell marker; B) and aromatase (pre-granulosa cell marker; C) was performed in transverse sections. Dashed box indicates the magnified area. Dotted line delineates the gonadal epithelium. White arrow indicates the epithelium (E) or the JCM. (D) Quantification of the average epithelium thickness (in μm) in control or TGIF1-overexpressing female gonads. (E) Quantification of the percentage of JCM area, relative to the total medullar area. Bars represent mean±s.e.m., *n*=3. Unpaired two-tailed *t*-test. *P<0.05.

To evaluate whether TGIF1 overexpression was able to rescue cortical regression, as occurs naturally in right female gonads, TOL2-TGIF1-OE or GFP control plasmids were electroporated into the right coelomic epithelium at E2.5 (HH stage 14). Embryos were collected at E8.5, PCR-sexed and immunofluorescence was performed for different gonadal markers. TGIF1 overexpression in the right ovary did not induce complete cortex formation (Fig. 6A). However, similar to the effect in left gonads, the epithelial cells misexpressing TGIF1 were thicker than those of the control in both female (Fig. 6A-D) and male (Fig. S5A-D) gonads, showing a cuboidal morphology. This indicates that TGIF1 alone does not induce complete cortex formation but may function to block the surface epithelial flattening that naturally occurs in the right ovary and male gonads. In addition, TGIF1 overexpression induced accumulation of fibronectin-positive cells in the juxtacortical region of the gonad of both sexes (Fig. 6B, Fig. S5B), resulting in an increase in the JCM area (Fig. 6E, Fig. S5E) and displacement of the supporting cells from the medullar region (Fig. 6C, Fig. S5C).

### Ovarian TGIF1 knockdown inhibits gonadal cortex and JCM formation

**Fig. 7.**
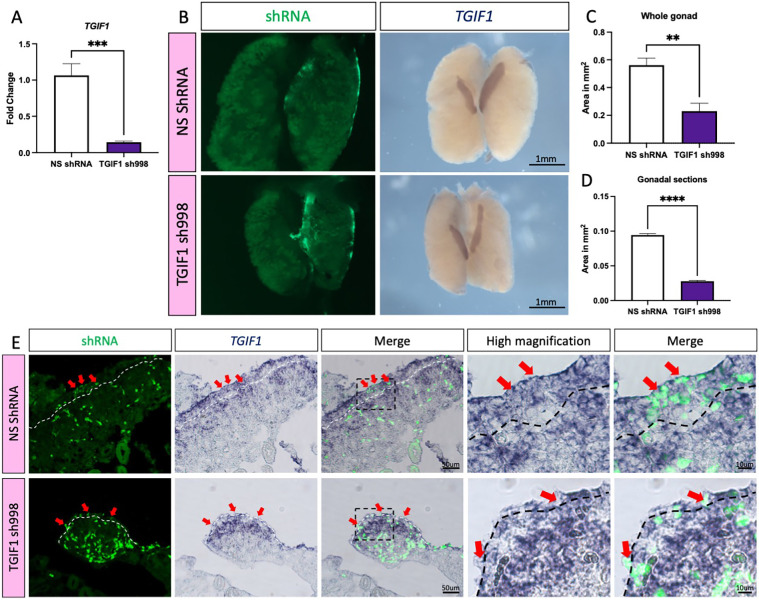
**TGIF1 knockdown results in smaller ovaries.** (A) DF1 cells expressing TGIF1 sh998 or non-silencing control (NS shRNA) were challenged for 48 h with RCAS(A)-GFP-T2A-TGIF1 overexpression plasmid. *TGIF1* mRNA was quantified by qRT-PCR. Expression level is relative to β-actin and normalized to NS shRNA (*n*=6). (B) TOL2 TGIF1 knockdown (TGIF1 sh998) or non-silencing control (NS shRNA) plasmids were co-electroporated with a GFP-expressing plasmid (reporter) in female left E2.5 coelomic epithelium. Gonads were examined at E8.5 for GFP expression and *TGIF1* whole-mount *in situ* hybridization was performed. (C) Quantification of the gonadal area (in mm^2^) from whole-mount images (*n*=5). (D) Quantification of the gonadal area (in mm^2^) from whole-mount gonadal sections (*n*=5). Bars represent mean±s.e.m. **adjusted *P*<0.01, ***adjusted *P*<0.001, ****adjusted *P*<0.0001. Unpaired two-tailed *t*-test. (E) Whole-mount sections (10 μm) were processed for immunofluorescence against GFP. Dashed white line delineates the gonadal epithelium. Red arrows indicate GFP-positive (shRNA-targeted) epithelial/cortical cells.

*In ovo* gene knockdown by RNA interference was performed to assess the role of TGIF1 during cortical and JCM formation in the developing ovary. Four different short hairpin RNAs (shRNAs) were designed against the *TGIF1* ORF and were cloned into the retroviral vector RCASBP(A) carrying a blue fluorescent protein (BFP) reporter. These were screened for knockdown efficiency *in vitro* using the DF1 chicken fibroblastic cell line. DF1 cells were transfected with plasmids carrying BFP-T2A and a non-silencing control shRNA (NS shRNA), or one of four different shRNAs designed for *TGIF1* knockdown. After all cells became BFP positive, they were challenged with TOL2 plasmid overexpressing chicken TGIF1-GFP (*TOL2-GFP-T2A-TGIF1*). Plasmid expressing mCherry was used as a transfection control. Forty-eight hours after transfection, cells were fixed and GFP fluorescence was quantified as a measure of *TGIF1* knockdown (Fig. S6A). All of the shRNAs showed a significant decrease of GFP intensity. *shRNA-998* showed the strongest inhibition (66%), followed by *shRNA-364* (57%), *shRNA-416* (46%) and *shRNA-318* (16%). These values were calculated using the mean of each group with NS shRNA as a control (100%) (Fig. S6B). Additionally, *shRNA-998* knockdown efficiency was evaluated in DF1 cells by qRT-PCR. DF1 cells expressing TGIF1 *shRNA-998* or NS shRNA were transfected with TGIF1 overexpression construct. Cells were collected 48 h post-transfection and *TGIF1* mRNA expression levels were quantified by qRT-PCR. *TGIF1 shRNA-998* significantly reduced *TGIF1* mRNA expression, resulting in 86.8% inhibition compared with the control (NS shRNA) (Fig. 7A).

To evaluate knockdown efficacy *in vivo*, *TGIF1 shRNA-998* was cloned into a TOL2 vector expressing nuclear BFP. *TOL2-TGIF1shRNA-998-nBFP* or *TOL2-NSshRNA-nBFP* were co-electroporated with *TOL2-CAGGS-GFP* (electroporation reporter) and transposase plasmid into the left coelomic epithelium at E2.5. Urogenital systems were collected at E8.5, genetically sexed by PCR and *TGIF1* WISH was performed (Fig. 7B). *TGIF1* knockdown resulted in a substantial size reduction of the targeted left ovaries (Fig. 7B-D). Additionally, *TGIF1* WISH staining appeared weaker in *shRNA-998*-expressing gonads, compared with the NS controls. In addition, *TGIF1* mRNA staining was weaker in the left targeted gonad compared with the right un-electroporated gonad (Fig. 7B). Immunofluorescence against GFP was performed on overstained whole-mount *in situ* sections to evaluate the colocalization of GFP (i.e. shRNA) and *TGIF1* mRNA. As expected, the controls exhibited normal ovarian histology, with *TGIF1* expressed in both the cortex and the medulla (Fig. 7E). GFP-positive cells in the cortex were also positive for *TGIF1* staining, showing that *TGIF1* mRNA was not knocked down in the controls. In contrast, ovaries treated with *TGIF1 shRNA-988* were smaller in size than the controls and exhibited a thinner cortex (Fig. 7D,E). Although electroporation is innately variable, causing mosaic DNA delivery, individual GFP^+^ (*shRNA-998*^+^) epithelial cells lacked *TGIF1* purple staining, confirming *TGIF1* knockdown *in vivo* (Fig. 7E). *TGIF* mRNA expression was still robust in the medulla of knockdown gonads, as expected, as this compartment was not targeted (Fig. 7E).

**Fig. 8.**
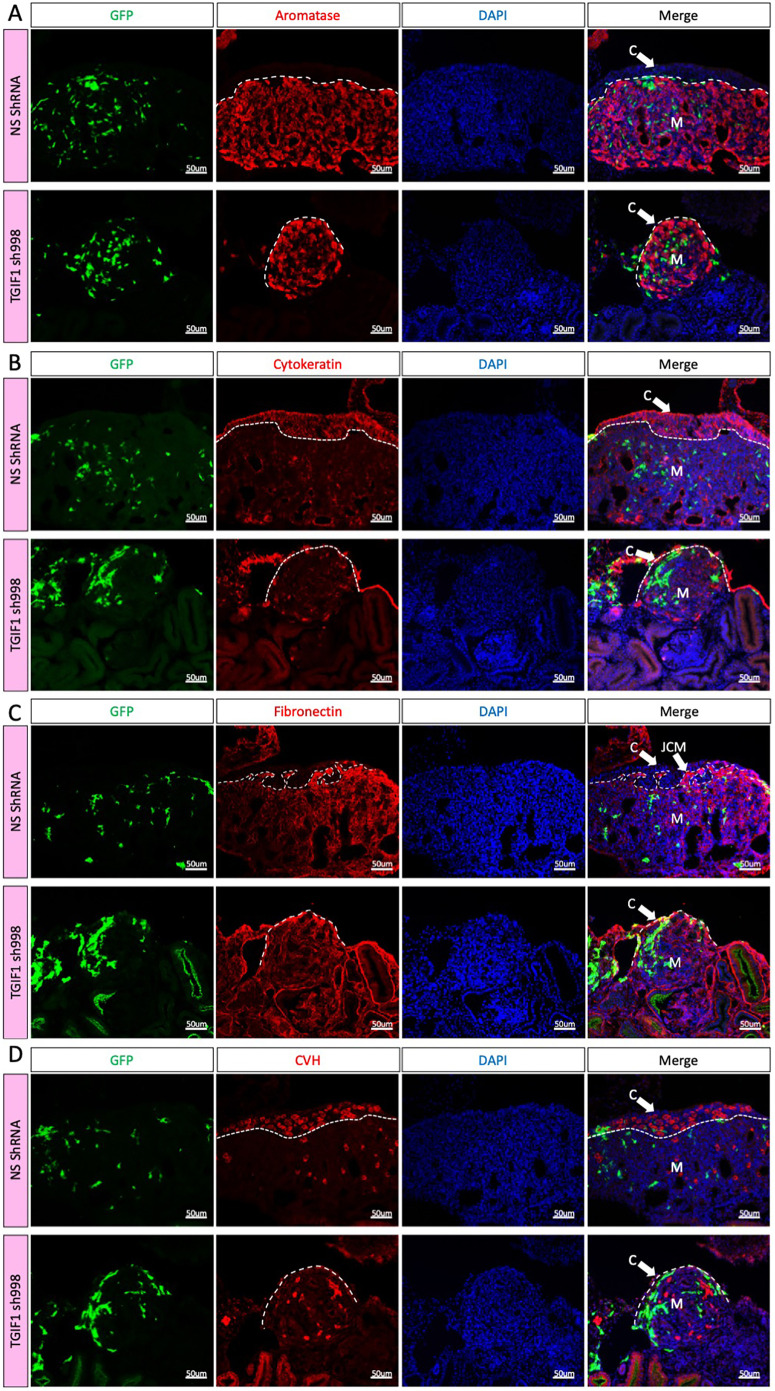
**TGIF1 knockdown ablates cortical and JCM formation in female gonads.** (A-D) TOL2 TGIF1 knockdown (TGIF1 sh998) or non-silencing control (NS shRNA) plasmids were co-electroporated with a GFP-expressing plasmid (reporter) in female left E2.5 coelomic epithelium. Gonads were examined at E8.5. Immunofluorescence against aromatase (pre-granulosa marker; A), cytokeratin (epithelial/cortical marker; B), fibronectin (interstitial cell marker; C) and CVH (germ cell marker; D) was performed on transverse sections. Dashed line delineates the gonadal epithelium. Arrows indicate the cortex (C) or the JCM. M indicates the medulla.

To evaluate the effect of *TGIF1* knockdown on gonadal morphology, immunofluorescence was performed for gonadal markers. Aromatase-positive pre-granulosa cells were still present in the gonadal medulla (Fig. 8A). Strikingly, an ovarian cortex was absent in the female *TGIF1* knockdown gonads, as revealed by greatly diminished cytokeratin expression (Fig. 8B). Instead, the epithelial cells exhibited a flattened morphology similar to the right gonadal epithelium or the testicular epithelium (Fig. 8B). In addition, these ovaries lacked a clear JCM, evidenced by the absence of condensed fibronectin-positive cells in between the epithelium and the aromatase-positive pre-granulosa cells (Fig. 8C). Owing to the absence of a defined cortex, the germ cells remained in the medulla (similar to their fate in the right gonad) (Fig. 8D). Sertoli cell markers (SOX9 and DMRT1) were not upregulated (Fig. S7). Altogether, these results indicate that TGIF1 is necessary for development of the ovarian cortex. Moreover, TGIF1 is the first gene reported to be required for proper JCM formation.

## DISCUSSION

Gonadal sex differentiation provides an ideal model for studying cell fate decisions during embryogenesis (Lin and Capel, 2015; Munger and Capel, 2012). During gonadal morphogenesis, cell lineages differentiate into ovarian or testicular cell types. The first cells to differentiate are the supporting cells (Sertoli cells in males, pre-granulosa cells in females) (Nef et al., 2019). These cells then signal to control differentiation of the other gonadal lineages, including steroidogenic and non-steroidogenic cells, and they also influence germ cell fate (spermatogonia in males, oogonia in females) (Stévant et al., 2019; Stévant and Nef, 2019). Given the central role of the supporting cell population, most research in the field has focused on the granulosa versus Sertoli cell fate decision. Less is understood about the development and role of the gonadal surface (coelomic) epithelium. However, lineage tracing and single-cell RNA-seq have shown that this gonadal compartment has significantly different roles in mammalian versus avian models. In the mouse embryo, the surface epithelium is central to gonadal differentiation. This layer of cells is the source of most somatic cell progenitors in the embryonic murine gonad (DeFalco et al., 2011; Nicol and Yao, 2014). Somatic cells of the surface epithelium in mouse divide asymmetrically, producing one daughter cell that remains at the surface and one that undergoes EMT, ingressing into the gonad. This process is regulated by Notch signaling, via the antagonist Numb (Lin et al., 2017). Homeobox transcription factors, such as Emx2, Six1 and Six4, contribute to this EMT (Kusaka et al., 2010; Fujimoto et al., 2013). In contrast to mouse, lineage tracing in the chicken embryo clearly shows that proliferating surface epithelium gives rise to non-steroidogenic interstitial cells, not the supporting cell lineage (which derives from mesonephric mesenchyme) (Sekido and Lovell-Badge, 2007; Estermann et al., 2020). Furthermore, epithelial cells differentiate into a stratified layer of cortical cells in the left female gonad, whereas this process does not occur in males (Estermann et al., 2020). The development of a thickened left gonadal cortex is crucial for proper ovary formation and female reproduction in birds. Germ cells accumulate in the ovarian cortex during embryonic stages and are signaled to enter meiotic prophase (Smith et al., 2008). After hatching, germ cell development proceeds as the cortex is the site of folliculogenesis in avians (Johnson and Woods, 2009; Li et al., 2016; Hu et al., 2021). The importance of the cortex is revealed by the asymmetry of female avian gonadal development. The right gonad fails to elaborate as cortex in females and germ cells remain in the medulla, where they eventually become atretic (Guioli et al., 2014).

The results presented here demonstrate that the TALE homeobox gene *TGIF1* plays a key role in development of the gonadal cortex in the chicken embryo. This gene is upregulated during female but not male gonadal development (Fig. 1). It is strongly expressed in the female gonadal surface epithelium at the onset of sexual differentiation (E6.5/stage 30), and in the gonadal medulla. Targeted overexpression in the male surface epithelium induces a thickened epithelium, whereas targeted knockdown in females blocks proper cortical layer development. Manipulation of expression in the medulla did not have an overt effect upon gonadogenesis. Furthermore, *TGIF1* expression was responsive to modulation of estrogen, which is essential for ovarian development in birds (Scheib, 1983; Elbrecht and Smith, 1992; Vaillant et al., 2001a). Inhibition of the estrogen-synthesizing enzyme aromatase resulted in downregulation of *TGIF1* expression (Fig. 4). This indicates that TGIF1 is a downstream target of estrogen, either directly or indirectly, during ovary formation. In the chicken embryo, two roles are ascribed to the estrogen that is synthesized by medullary cords cells of female embryos at the onset of gonadal sex differentiation. Firstly, estrogen acts in the medulla itself to antagonize the induction of the testis factors DMRT1 and SOX9 (Smith et al., 2003; Ioannidis et al., 2021). Secondly, it acts on the surface epithelium in a paracrine fashion, where it stimulates development of the gonadal cortex (Gasc and Stumpf, 1981; Wartenberg et al., 1992). Correspondingly, estrogen receptor α (ERα) is expressed in both gonadal compartments in chicken embryos, though more strongly in the cortex (Andrews et al., 1997; González-Morán, 2014). Exogenous estrogens can induce cortical cell differentiation in embryonic male (ZZ) gonads (Guioli et al., 2020). Whereas gonadal asymmetry in the chicken is driven by asymmetric expression of *PITX2* in the cortex (Rodríguez-León et al., 2008; Guioli and Lovell-Badge, 2007), cortical cell proliferation is related to estrogen action. ERα is expressed in the left but not the right gonadal epithelium. This is consistent with the cortical development in the left but not in the right gonad. RNAi or dominant negative-mediated downregulation of ERα cause a reduction in cortical size, indicating that ERα and estrogens are essential for cortex formation (Guioli et al., 2020).

Here, we found that *TGIF1* expression was induced by estrogen and downregulated when estrogen synthesis was inhibited (Figs 3 and 4). Moreover, *TGIF1* and ERα are both expressed in the left gonadal epithelium (and in supporting cells of the medulla), suggesting that estrogens, through ERα, could regulate *TGIF1* expression in chicken ovaries. This would also explain why *TGIF1* is expressed in the left gonadal epithelium but not in the right (Fig. 2). Similar to ERα, TGIF1 knockdown in female gonads resulted in lack of cortical development, despite the presence of estrogens (aromatase expression was not perturbed). This indicates that *TGIF1* is required for ovarian cortical formation, acting downstream of the estrogen signaling pathway. It will be of interest to examine the regulatory region of the chicken *TGIF1* gene for estrogen response elements. However, although misexpression of TGIF1 in male gonads blocked epithelial flattening and induced thickening, a complete female-like cortex was not induced. This indicates that other factors are also required, one of which is likely to be estrogen, which is absent in male gonads. Hence, estrogen may activate TGIF1 expression and then function together with this factor to direct complete cortex formation. It would be of interest to study the effects of TGIF1 upon epithelial cell proliferation and on cyclin expression (the latter being a known target of estrogen signaling).

The data presented here show that loss of TGIF1 prevents proper cortex development in female gonads, and misexpression in males causes thickening of the surface epithelium but not complete cortex formation. Taken together, these results indicate that *TGIF1* is necessary but not sufficient for ovarian cortex formation in chicken. One of the functions of TGIF1 appears to be the maintenance of columnar epithelial cells in the gonadal cortex. The epithelium in the left and right gonads in both males and female chicken embryos at E6.5 shows an asymmetry, being thicker in the left than the right gonad (Guioli et al., 2014). In females, this structure continues proliferating, whereas in the male it flattens to form a squamous monolayer. *TGIF1* overexpression in male (left and right) and female (right) gonads did not induce the formation of a multilayered female-like cortex. However, epithelial cell thickness was increased (Figs 5 and 6, Figs S3 and S5). This suggests that TGIF1 is required for maintaining columnar epithelial structure and inhibiting a squamous phenotype. *Tgif1*/*Tgif2* double-null mouse embryos display disorganized epiblasts, which lack the typical columnar epithelial morphology (Powers et al., 2010). This suggests that the role of *TGIF1* in maintaining the epithelium structure may be a conserved function during embryogenesis.

*TGIF1* may be acting through a number of mechanisms to promote development of the ovarian cortex in the chicken embryo. At least three signaling pathways have been linked to TGIF1 function: TGF (Wotton et al., 1999b), retinoic acid (Bertolino et al., 1995) and Wnt/β-catenin (Zhang et al., 2015b). All of these pathways are known to be engaged in the embryonic gonads in chicken and in mouse. TGIF1 is a TGFβ signaling inhibitor, binding to phospho-Smad2, recruiting histone deacetylases and acting as a co-repressor of Smad target genes (Wotton et al., 1999a). Chicken ovaries exposed to TGFβ1 display a reduction of somatic cells due to decreased cell proliferation (Méndez et al., 2006). In addition, there is a reduction in the number of germ cells in the cortex and an increased number in the medulla (Méndez et al., 2006).This suggests an effect in the cortical compartment or in the capacity of germ cells to migrate. In mice, nodal, activin and TGFβ signal through Smad2/3/4 and are important for testicular development, suppressing the pre-granulosa program (Gustin et al., 2016; Wu et al., 2013). In contrast, BMP molecules, such as BMP2, signal through Smad1/5/8 and are important for ovarian differentiation in mouse (Kashimada et al., 2011). TGIF1, being expressed in the female supporting cells and cortex, could act to repress the Smad2/3 masculinizing signaling, thus allowing BMPs to participate in ovarian differentiation. Among the BMP proteins, *BMP7* has been shown to be asymmetrically expressed in undifferentiated chicken left gonads, making it a good candidate for further research in the context of TGIF1 (Hoshino et al., 2005).

It has been shown that the conserved transcription factor, PITX2, mediates left-right asymmetry and development of the thickened left gonadal cortex in chicken embryos. PITX2 is endogenously expressed only in the left gonad, and misexpression in the right can rescue its programmed degeneration, producing a well-developed cortex. (Rodríguez-León et al., 2008; Guioli and Lovell-Badge, 2007; Ishimaru et al., 2008). Using gene overexpression and domain-negative approaches *in ovo*, Ishimaru et al. showed that left-restricted early expression of PITX2 blocks retinoic acid function, allowing Ad4BP/Steroidogenic Factor 1 to stimulate cyclin D1 and cortical cell proliferation, and also allowing ERα expression. In the right gonad, in the absence of PITX2, retinoic acid blocks SF1, cell proliferation and ERα expression. TGIF1 is likely to operate downstream of ERα in this cascade, given the later timing of its induction and its response to E2 manipulation. We found that misexpression of TGIF1 in male gonads induces surface epithelial thickening, but not complete cortex formation, most likely due to the absence of estrogen as noted above. In right female gonads where TGIF1 was misexpressed, we noted the same epithelial thickening, but again no cortex formation, in this case most likely owing to the absence of functional ERα in the right epithelium (Andrews et al., 1997; Guioli et al., 2014; Guioli and Lovell-Badge, 2007). The partial thickened cortical phenotype also be due to the presence of endogenous retinoic acid synthesis in the right gonad, which has been shown to antagonize cortical proliferation, as noted above (Ishimaru et al., 2008).

The data presented here indicate that TGIF1 also plays a role in JCM formation. The JCM represents an accumulation of interstitial cells directly underneath the cortex in females. This structure is not present in testes, and its functional significance is not known. However, several genes show restricted expression in the JCM later in development, such as *CYP26B1*, which is responsible for retinoic acid degradation (Smith et al., 2008). Although *TGIF1* is not expressed in the JCM (Figs 1D and 2), it is expressed in the surface epithelial cells that we know from previous lineage tracing give rise to the JCM and other interstitial cells (Estermann et al., 2020). Misexpression of TGIF1 in male gonads caused areas of ectopic JCM (Fig. 5C). This indicates that TGIF1 expression in the surface epithelium can drive underlying JCM formation. Conversely, knockdown of epithelial TGIF1 expression in female abolishes JCM formation (Fig. 8). Together, the data indicate that asymmetric cell division in the female gonadal cortex and EMT to generate the JCM are at least partly regulated by TGIF1.

Previously, it has been shown that asymmetric cell division in the chicken left cortex (parallel to the epithelial plane) is twice that of the right (Rodríguez-León et al., 2008). Such asymmetric division is associated with epithelial stratification and production of new cell types that leave the epithelium (Woolner and Papalopulu, 2012). TGIF1 may play some role in this process, and it will be of interest to study both asymmetric cell division and the expression of EMT markers after TGIF1 misexpression. Indeed, over recent years TGIF1 has been shown to be involved in EMT in several cell types, tissues and organisms. TGIF1 is expressed in chicken dorsal neural tube and in delaminating cardiac neural crest, where it is required for the formation of mesenchymal derivatives of the crest (Gandhi et al., 2020). In addition, TGIF1 is associated with increased breast, lung and colorectal cancer migration and metastasis (Haider et al., 2020; Xiang et al., 2015; Wang et al., 2017; Zhang et al., 2015b). The data presented here are consistent with these previous reports. TGIF1 overexpression in male coelomic epithelium induced fibronectin-positive interstitial (mesenchymal) cell accumulation underneath the gonadal epithelium (Fig. 5, Fig. S5). Consistent with this, ovaries lacked a JCM when TGIF1 was knocked down (Fig. 8). TGIF1 is the first gene reported to be required for JCM formation. Moreover, this process appears to be independent of estrogen signaling, as shown by the absence of aromatase expression, and, consequently, estrogens in the male gonads misexpressing TGIF1 or ERα in right gonads.

TGIF1 was also expressed in the pre-granulosa cells, colocalizing with aromatase (Fig. 2). This suggest that TGIF1 could play a role in supporting cell differentiation. SOX9 is a marker of Sertoli cells, and is known to have a role in repressing the female differentiation pathway and inducing and maintaining the male genetic program, at least in mammals. When Tgif1 was overexpressed in mouse limb mesodermal micromass cultures, chondrogenic markers, such as Sox9, were downregulated (Lorda-Diez et al., 2009). When Tgif1 was silenced, Sox9 expression was upregulated, suggesting a direct or indirect role of Tgif1 in repressing Sox9 (Lorda-Diez et al., 2009). Here, overexpression of TGIF1 in testicular supporting cells (via the RCASBP virus) did not result in reduction of SOX9 expression or upregulation of pre-granulosa markers (Figs S3 and S4). This suggests that this role of TGIF1 is not conserved between mouse and chicken and that its function differs between limbs and gonads. The current data presented here indicate that TGIF1, by itself, has no role in the early differentiation of supporting cells in the chicken model.

TGIF1 and TGIF2 share similar spatial and temporal expression during embryonic development. In addition, they have similar binding domains, suggesting functional redundancy (Shen and Walsh, 2005; Powers et al., 2010). TGIF2 has redundant functions with TGIF1, but as transcription factors they must be co-expressed in the same cells in order to have a compensatory effect (Lee et al., 2015). In chicken gonads, both TGIF1 and TGIF2 are expressed in the gonads, but RNA-seq data showed that *TGIF2* mRNA expression is lower than *TGIF1* expression (Fig. 1A, Fig. S1A). In ovarian *TGIF1* knockdown experiments, endogenous *TGIF2* expression was not able to rescue cortical and juxtacortical formation. This suggests that in chicken gonads TGIF1 and TGIF2 do not share exactly the same functions, or that they are not expressed in the same cell types. Although TGIF1 has not previously been linked to vertebrate gonadal development, *Drosophila* TGIF and tammar wallaby TGIF2 are important for spermatogenesis (Ayyar et al., 2003; Hu et al., 2011). In mouse gonadal bulk RNA-seq data sets, *Tgif2* did not show any dimorphic expression (Zhao et al., 2018), similar to the chicken data (Fig. S1). Given the reported role in spermatogenesis, it would be interesting to study TGIF2 expression in adult birds to determine whether this function is conserved among species. *Tgif1* expression, by contrast, was sexually dimorphic in mice at E13.5, being highly expressed in males (Zhao et al., 2018). Further research would be required to evaluate the role of *Tgif1* in mammalian gonadal differentiation and development.

**Fig. 9.**
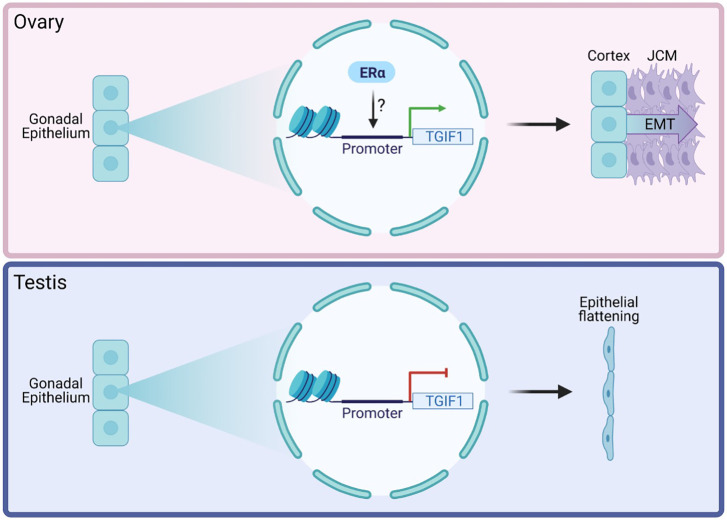
**Role of TGIF1 in epithelial maintenance and JCM formation.** In chicken ovaries (top), activation of the ERα signaling pathway by estrogens induces (directly or indirectly) the expression of TGF1 in the gonadal epithelium, resulting in epithelial structure maintenance and formation of the JCM by increased EMT. In chicken testis (bottom), TGIF1 expression is not induced in the gonadal epithelial cells due to the lack of ERα signaling. This results in the epithelial flattening and the lack of JCM formation.

In summary, in chicken ovaries, the data presented here indicate that activation of the ERα signaling pathway by estrogens induces (directly or indirectly) expression of *TGIF1* in the gonadal epithelium of the female chicken embryo. TGIF1 expression supports the development of the ovarian cortex, inhibiting epithelial flattening, and it induces formation of the JCM by increased EMT (Fig. 9). In chicken testis, TGIF1 expression is not induced in the gonadal epithelial cells because of the lack of estrogens and, consequently, ERα signaling. This results in epithelial flattening, inhibiting the formation of the JCM (Fig. 9). Forced expression of *TGIF* in male gonads (and in right female gonads) results in a thickened surface epithelium but not complete cortex formation. This shows that TGIF is necessary but not sufficient for gonadal cortex formation. Our results support the proposal that supporting cell differentiation and cortical sex differentiation are two independent processes (Guioli et al., 2020; Ioannidis et al., 2021). This research introduces TGIF1 as a regulator of gonadal cortex differentiation. In addition, we identified TGIF1 as the first known regulator required for JCM formation and provide evidence that a fully developed cortex or estrogens are not required for this process. Future research should focus on the downstream targets of TGIF1 in regulating these processes. In addition to its role as a transcription factor, TGIF1 has also been associated with several functions in signaling pathways. These include the TGFβ, retinoic acid and WNT/β-catenin pathways (Lorda-Diez et al., 2009; Liu et al., 2014; Gongal and Waskiewicz, 2008; Castillo et al., 2010; Zhang et al., 2015b). A comprehensive analysis of the role of TGIF1 in regulating cell signaling in the gonadal context is required to understand fully its role in gonadogenesis. This could also shed light on the role of TGIF1 in the supporting cell, which still remains unknown. Our research provides new insights into chicken ovarian differentiation and development, specifically in the process of cortical and JCM formation.

## MATERIALS AND METHODS

### Eggs and samples

Fertilized HyLine Brown chicken (*Gallus domesticus*) eggs were obtained from Research Poultry Farm (Victoria, Australia) and incubated under humid conditions at 37°C. Gonads and urogenital systems were collected at various time points throughout development and staged *in ovo* according to Hamburger and Hamilton (1951). PCR sexing was performed as described previously (Clinton et al., 2001).

### qRT-PCR

Gonadal pairs were collected in Trizol reagent (Thermo Fisher Scientific) and kept at −80° until processing. After sexing, three gonadal pairs from the same sex were pooled for each sample, homogenized and RNA extraction was carried out using the phenol-chloroform method as per the Trizol manufacturer's instructions. Genomic DNA was removed from the RNA samples using the DNA-free DNA Removal Kit (Invitrogen) and 200-500 ng of RNA was converted into cDNA using the Promega Reverse Transcription System. qRT-PCR was performed using the QuantiNova SYBR Green PCR Kit. Expression levels were quantified by the Pfaffl method (Pfaffl, 2001) using β-actin as housekeeping gene. Data were analyzed using multiple unpaired, parametric, two-tailed *t*-tests (one per embryonic stage or treatment). Statistical significance was determined using the Holm–Sidak method. Primers are listed in Table S1.

### WISH

WISH was performed as previously described (Estermann et al., 2020). At least three embryos were used for each stage and sex. Urogenital systems were dissected and fixed overnight in 4% paraformaldehyde. Tissues were dehydrated in a methanol series and stored until use. Samples then were rehydrated to PBS plus 0.1% Triton X-100 before digestion in proteinase K (1 μg/ml in PBS plus 0.1% Triton X-100) for 30 to 90 min at room temperature. Tissues were then washed, briefly refixed, and incubated overnight at 65°C in (pre)hybridization buffer. Digoxigenin-labeled antisense RNA probes were synthesized using a digoxigenin labeling kit, according to the manufacturer's instructions (Life Technologies). TGIF1 probes (514 bp) were cloned from gonadal cDNA using the primers listed in Table S1. DNA sequences were cloned into the pGEM-T Easy vector and sequences were confirmed before use. For probe generation, a DNA template was first generated by PCR amplification of the insert, using M13 forward and reverse primers, encompassing RNA polymerase-binding sites. Antisense and sense digoxigenin-labeled RNA probes were generated using the relevant T7 or SP6 RNA polymerase sites present in the amplified PCR product. Following synthesis, riboprobes were precipitated overnight at −20°C. For each probe, 7.5 μl were added to 2 ml (pre)hybridization mix and incubated overnight at 65°C. Following low and high stringency washes, tissues were washed, preblocked, and incubated overnight at 4°C with alkaline phosphatase-conjugated anti-digoxigenin antibodies (1:2000; 11093274910, Roche) in TBTX [50mM Tris-HCl (pH 7.5), 150mM NaCl, 0.1% Triton X-100]. Following extensive washing in TBTX, tissues were exposed to chromogen (NBT/BCIP) for up to 3 h. For each gene, the color reaction was stopped at the same time by rinsing in NTMT buffer [100mM NaCl, 100mM Tris-HCl (pH 9.5), 50mM MgCl_2_, 0.1% Tween-20], followed by washing in PBS and imaging. Tissues were then overstained, cryoprotected in PBS plus 30% sucrose, snap-frozen in OCT embedding compound, and cryosectioned at 14-18 μm, or 10 μm if they were processed for immunofluorescence.

### Sex reversal

For masculinization, eggs were either injected with 1 mg of fadrozole (Novartis) in 100 μl of PBS or injected with PBS alone at E3.5 as previously described (Hirst et al., 2017a). For feminization, 17β-estradiol (Sigma-Aldrich) was initially resuspended in 100% ethanol (10 mg/μl) and then diluted to 1 mg/ml in sesame oil, and 100 μl of this 1 mg/ml solution (0.1 mg of E2) or 10% ethanol in sesame oil solution (vehicle) was injected into E3.5 eggs. Eggs were incubated until day 9.5 of development (HH34) before processing them for WISH.

### TGIF1 overexpression construct design and electroporation

The Tol2 system was used to integrate TGIF1 overexpression construct into the genome of electroporated cells in the chicken embryos (Kawakami, 2007; Sato et al., 2007). The TGIF1 ORF was amplified from gonadal cDNA using specific primers (see Table S1) and Gibson-cloned into TOL2-CAGGS-GFP or RCAS(A)-GFP and sequenced. DF1 cell (ATCC) transfection with TOL2- CAGGS-GFP-T2A-TGIF1 overexpression plasmid or control plasmid and transposase-expressing plasmid was performed following the Lipofectamine 2000 protocol (Life Technologies). Cells were collected 48 h post-transfection and Trizol RNA extraction was performed as described above.

*In ovo* electroporation of p-CAGGS-Transposase with TOL2-CAGGS-GcT-T2A-TGIF1 (TGIF1 OE) or TOL2-CAGGS-GFP (GFP control) constructs was performed as previously described (Hirst et al., 2017b) on E2.5 embryos, targeting the left coelomic epithelium. Embryos were harvested at E7.5-8.5, sexed and processed for immunofluorescence. For RCAS electroporation, RCAS(A)-GFP-T2A-TGIF1 (TGIF1 OE) or RCAS(A)-GFP (GFP control) were electroporated.

### TGIF1 shRNA design, validation and electroporation

TGIF1 shRNA design, validation and cloning was performed as previously described (Roly et al., 2020; Major et al., 2019). Four different shRNAs were designed against the TGIF1 ORF, ranked for effectiveness (Clarke et al., 2017) and cloned into the RCAS(A)-BFP plasmid. A PCR-based amplification of the shRNA template along with the chicken U6-4 promoter was used (Lambeth et al., 2015) (Table S1). Their ability to knock down TGIF1 expression was assessed *in vitro* in chicken fibroblastic DF1 cells. Firstly, DF1 cells were transfected with the plasmids containing BFP-T2A and a non-specific shRNA (firefly sh774) (Roly et al., 2020) or with one of four different putative shRNAs designed for TGIF1 knockdown (sh318, sh364, sh416 and sh998), following the Lipofectamine 2000 protocol (Life Technologies). Once all cells were BFP positive, they were transfected with the TOL2-GFP-T2A-TGIF1 overexpression plasmid, a transposase-expressing plasmid and a TOL2 plasmid expressing mCherry (as a transfection control) following the Lipofectamine 2000 protocol (Life Technologies). Forty-eight hours after transfection, cells were fixed in 4% paraformaldehyde for 15 min, stained with DAPI and imaged using a Leica AF600LX microscope. GFP-T2A-TGIF1 intensity was determined on a per cell basis using an established image analysis pipeline (Major et al., 2017). DAPI was used to identify the cell nuclei and mCherry-positive cells were gated for further analysis. To assess the ability of shRNA-998 to knock down TGIF1, T25 flasks containing DF1 cells were transfected with RCAS(A)-BFP-TGIF1-Sh998 or RCAS(A)-BFP-Firefly-Sh774. Seventy-two hours after transfection, they were collected using trypsin, and seeded in 24-well plates. After 24 h of resting, DF1 cells were transfected with RCAS(D)-GFP-T2A-TGIF1 plasmid. Forty-eight hours later, cells were collected and processed for RNA extraction as mentioned above. For *in vivo* experiments, TGIF1 sh998 was cloned into a TOL2 vector expressing nuclear BFP. TOL2-TGIF1 sh998-BFP or TOL2-Firefly sh774-BFP (non-silencing shRNA) was *in ovo* co-electroporated with a plasmid expressing transposase and TOL2-CAGGS-GFP (electroporation reporter) into the left coelomic epithelium at E2.5. Urogenital systems were collected at E8.5, sexed and *TGIF1* WISH or immunofluorescence against different gonadal markers was performed.

### Immunofluorescence

At least three embryos per time point and/or treatment were examined. Tissues were fixed in 4% paraformaldehyde/PBS for 15 min at room temperature. Tissues were cryoprotected in PBS plus 30% sucrose, snap-frozen in OCT embedding compound, and sectioned at 10 µm thickness. Some slides were first subjected to antigen retrieval, using an automated system, the Dako PT Link. Slides were firstly baked at 60°C for 30 min. Retrieval was then performed with the Dako Target retrieval solution (citrate-based, pH 6.0). Slides were then placed in the retrieval machine and retrieved at 98°C for 30 min. All sections were permeabilized in PBS containing 1% Triton X-100, blocked in PBS 2% bovine serum albumin (BSA) for 1 h, and incubated overnight at 4°C with primary antibodies in 1% BSA in PBS. Primary antibodies used were: goat anti-GFP (Rockland 600-101-215, 1:500), mouse anti-pan-cytokeratin (Novus Biologicals NBP2-29429, 1:200), rabbit anti-DMRT1 (in-house antibody, RRID: AB_2665399, 1:2000; Smith et al., 2003), rabbit anti-SOX9 (Millipore AB5535, 1:4000), rabbit anti-AMH (Abbexa ABX132175, 1:1000), rabbit anti-aromatase (in-house antibody, RRID: AB_2734780, 1:5000; Smith et al., 2003), mouse anti-fibronectin (Serotec 4470–4339, 1:500) and rabbit anti-CVH (in-house antibody, 1:500; Lambeth et al., 2013). Alexa Fluor secondary antibodies were used (donkey or goat anti-rabbit, mouse or goat 488 or 594; Life Technologies). Sections were counterstained with DAPI and mounted in FluorSave (Millipore). For WISH samples, sections were processed for antigen retrieval (as mentioned above). After the secondary antibody incubation, sections were treated with 0.3% Sudan Black (w/v) in 70% ethanol (v/v) for 10 min followed by eight quick PBS washes. Sections were counterstained with DAPI and mounted.

### Image quantification

Gonadal, epithelial, medulla and juxtacortical medulla area were manually quantified using Fiji (Schindelin et al., 2012). Epithelial average thickness was calculated by dividing the epithelial area by the length of the epithelium.

## Supplementary Material

Supplementary information

Reviewer comments
