# Supplementary material for: The homeobox gene TGIF1 is required for chicken ovarian cortical development and generation of the juxtacortical medulla
Source: Development. 2021 Aug 23;148(16):dev199646. doi: 10.1242/dev.199646 (PMC8406534; doi:10.1242/dev.199646)
Supplement: Supplementary information [file develop-148-199646-s1.pdf]

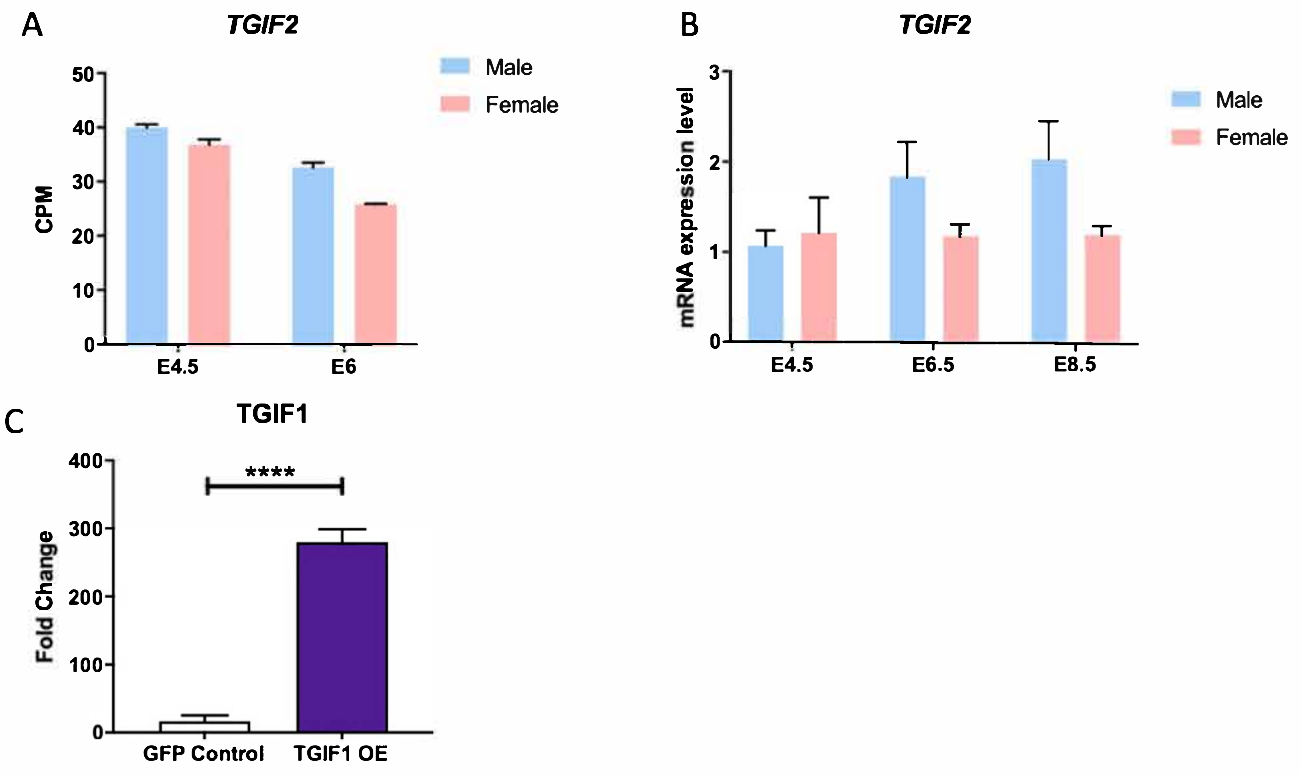

Fig. S1. ***TGIF2*** **expression is not sexually dimorphic in chicken gonads.** (A) *TGIF2* gonadal RNA-seq mRNA expression levels in count per million (CPM) at blastoderm stage, before (E4.5) and on the onset (E6) of sex determination. (B) *TGIF2* gonadal mRNA was quantified by qRT-PCR. Expression level is relative to β-actin and normalized to E4.5 male. (C) *TGIF1* mRNA was quantified by qRT-PCR in DF1 cells transfected with TGIF1 overexpression or GFP control plasmids. Expression level is relative to β-actin and normalized to GFP control. Bars represent Mean ± SEM. Unpaired two-tailed t-test. **** = p value <0.0001.

Development • Supplementary information

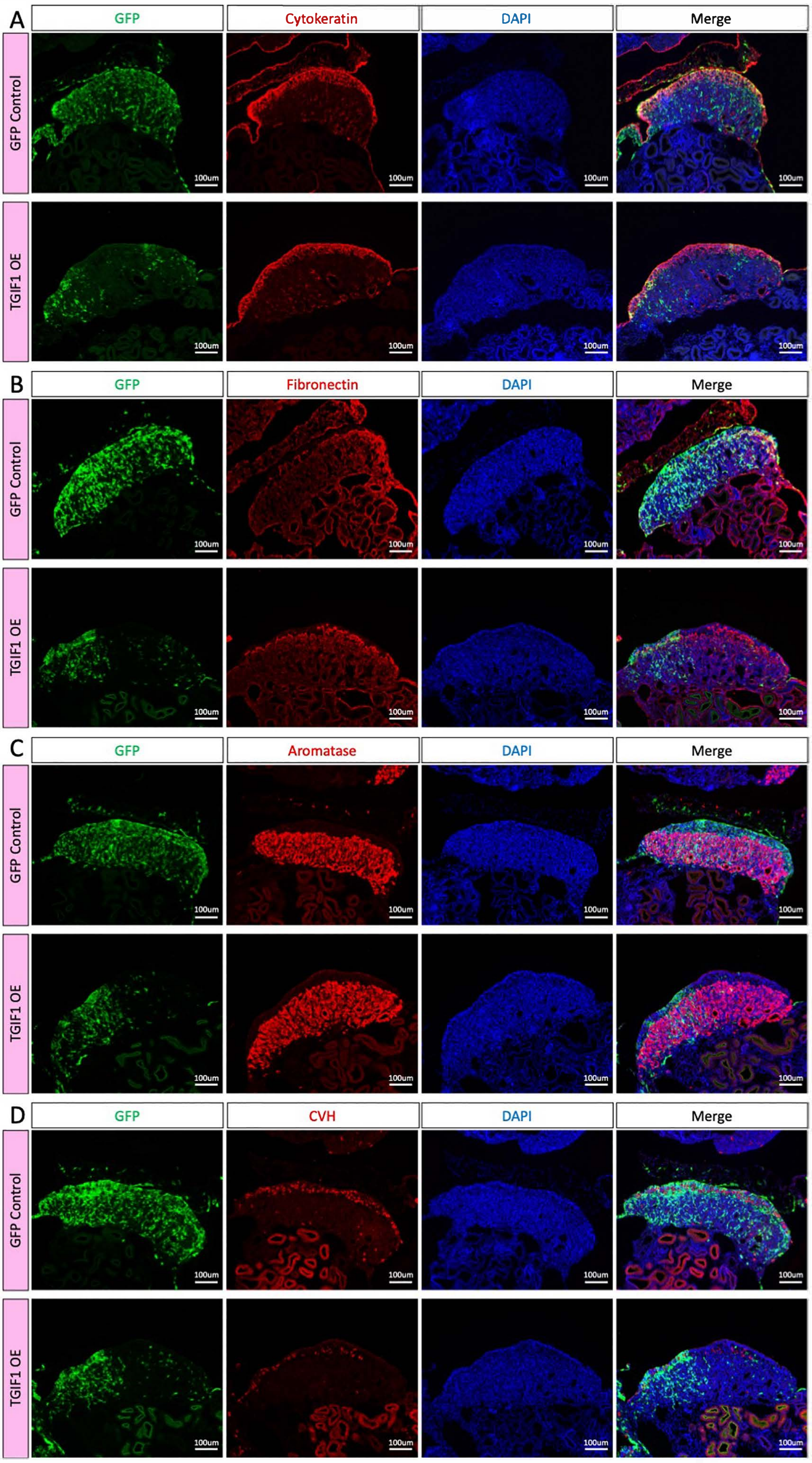

Development • Supplementary information

Fig. S2. TGIF1 overexpression in female left gonads has no effect. TOL2 TGIF1 overexpression (TGIF1 OE) or control (GFP OE) plasmids were electroporated in female left E2.5 coelomic epithelium. Gonads were examined at E8.5. Immunofluorescence against (A) cytokeratin (epithelial/cortical marker), (B) fibronectin (interstitial cell marker) and (C) aromatase (pre-granulosa cell marker) and (D) CVH (germ cell marker) were performed in transverse sections.

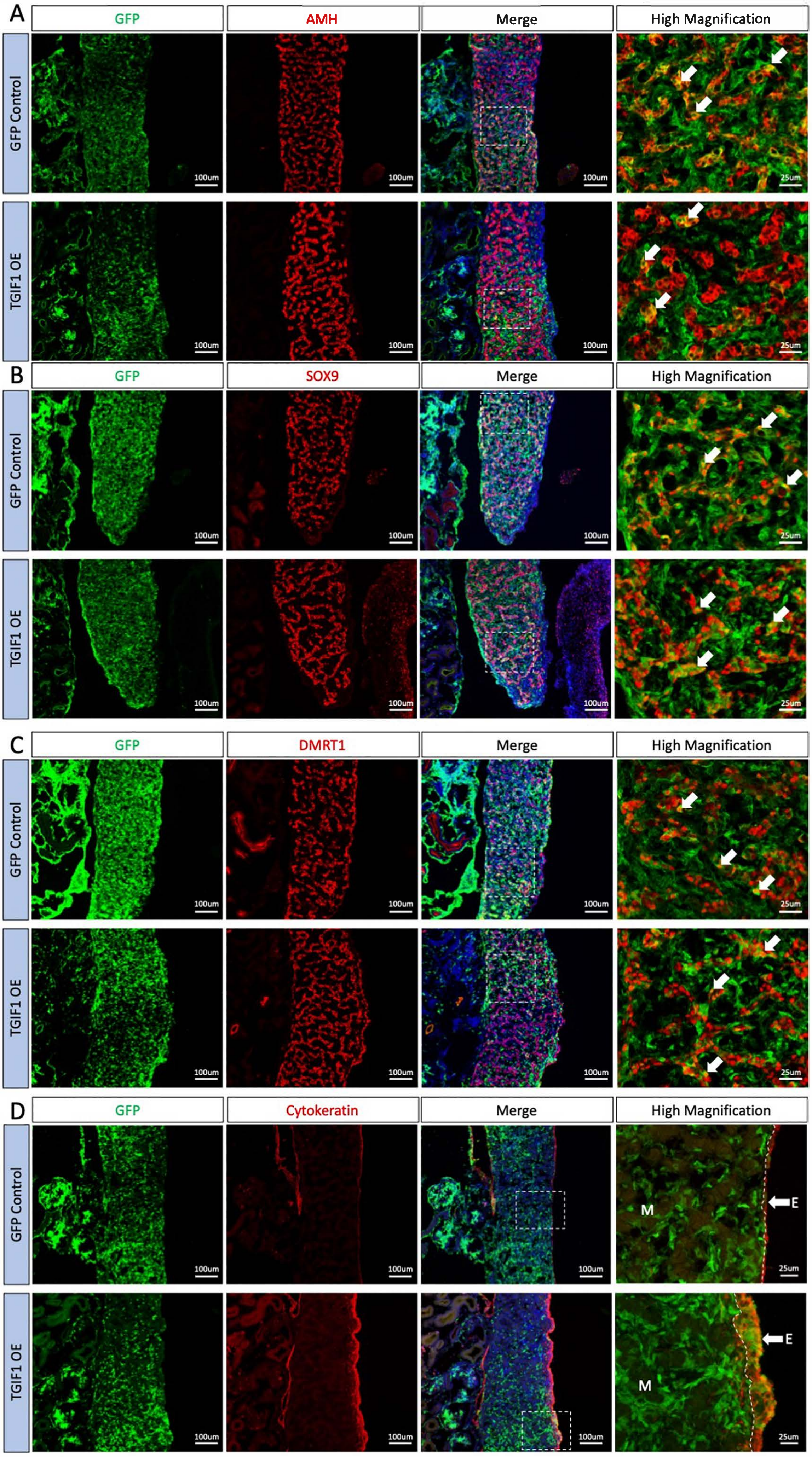

Development • Supplementary information

Fig. S3. **TGIF1 overexpression did not alter the supporting cell differentiation program.** RCAS(A) TGIF1 overexpression (TGIF1 OE) or control (GFP OE) plasmids were electroporated in male E2.5 left coelomic epithelium. Male gonads were examined at E8.5. Immunofluorescence against the supporting cell markers (A) AHM (B) SOX9 and (C) DMRT1. Dashed box indicates the magnified area. White arrows indicate colocalization of GFP (TGIF1) and Sertoli cell markers. (D) Immunofluorescence against the epithelial/cortical marker cytokeratin was performed in longitudinal male gonadal sections. Dashed box indicates the magnified area. Dashed line delineates the gonadal epithelium. White arrow indicates the epithelium (E). M indicates the medulla.

Development • Supplementary information

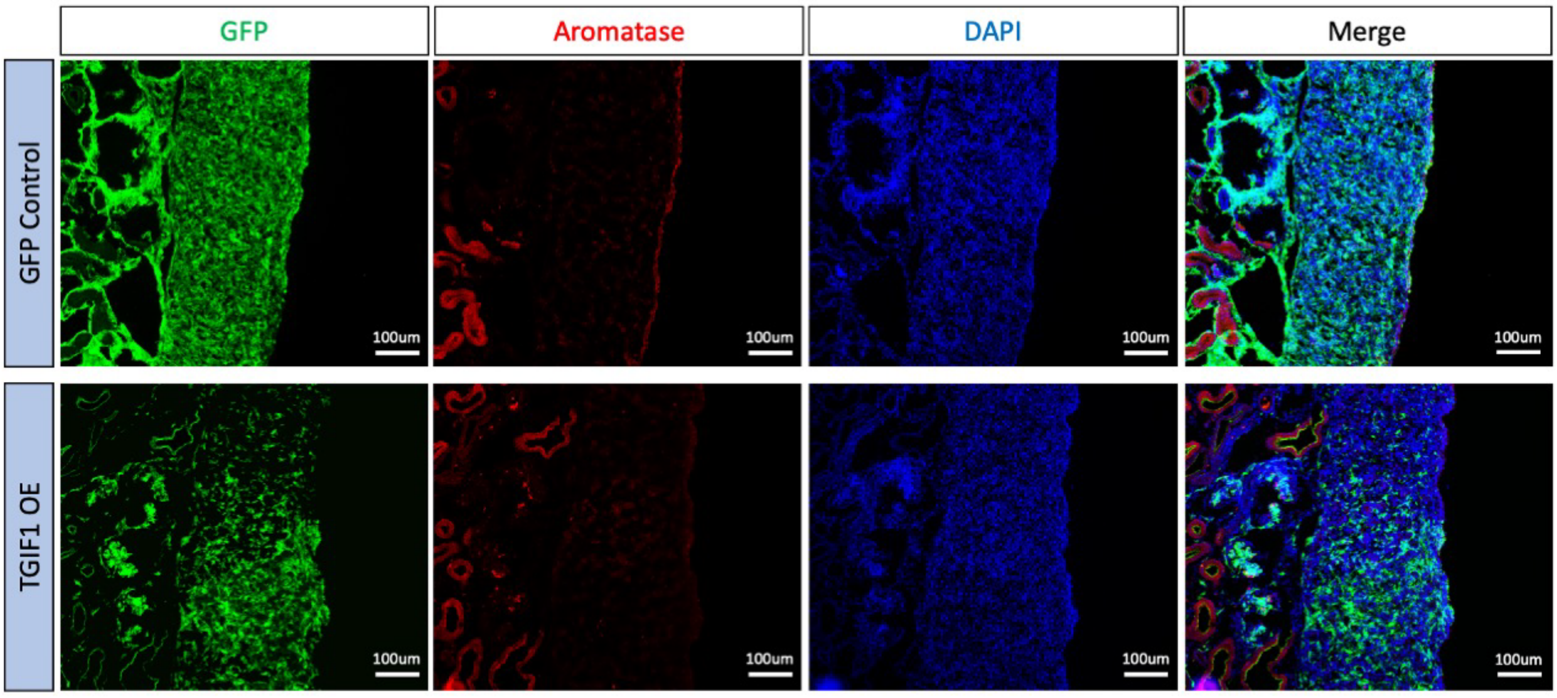

Fig. S4. **TGIF1 overexpression did not upregulate aromatase expression.** RCAS(A) TGIF1 overexpression (TGIF1 OE) or control (GFP OE) plasmids were electroporated in male E2.5 left coelomic epithelium. Male gonads were examined at E8.5. Immunofluorescence against the pre-granulosa markers aromatase was performed in longitudinal male gonadal sections.

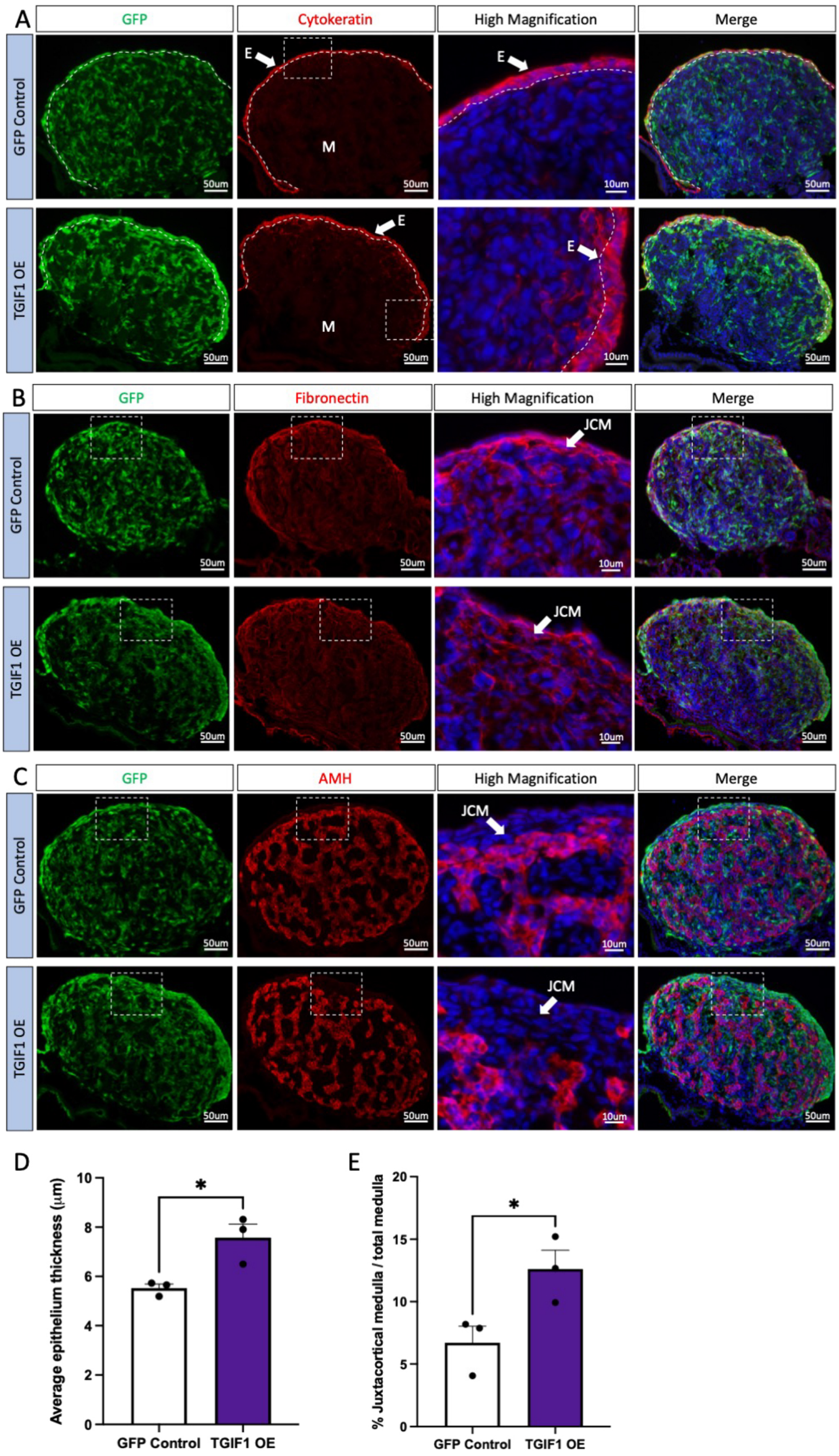
A
GFP
Cytokeratin
High Magnification
Merge
GFP Control
TGIF1 OE
E
M
50um
10um
B
GFP
Fibronectin
High Magnification
Merge
JCM
C
GFP
AMH
High Magnification
Merge
JCM
D
Average epithelium thickness (µm)
*
GFP Control
TGIF1 OE
E
% Juxtacortical medulla / total medulla
*
GFP Control
TGIF1 OE

Fig. S5. **TGIF1 overexpression in right male gonads results in epithelial thickening and JCM formation.** TOL2 TGIF1 overexpression (TGIF1 OE) or control (GFP Control) plasmids were electroporated in male right E2.5 coelomic epithelium. Gonads were examined at E8.5. Immunofluorescence against (A) cytokeratin (epithelial/cortical marker), (B) fibronectin (interstitial cell marker) and (C) AMH (Sertoli cell marker) were performed in transverse sections. Dashed box indicates the magnified area. Dotted line delineates the gonadal epithelium. White arrow indicates the epithelium (E) or the juxtacortical medulla (JCM). (D) Quantification of the average epithelium thickness (in um) in control or TGIF1 overexpressing male gonads. (E) Quantification of the percentage of juxtacortical medulla area, related to the total medullar area. Bars represent Mean ± SEM, n=3. Unpaired two-tailed t-test. * = p value <0.05.

Development • Supplementary information

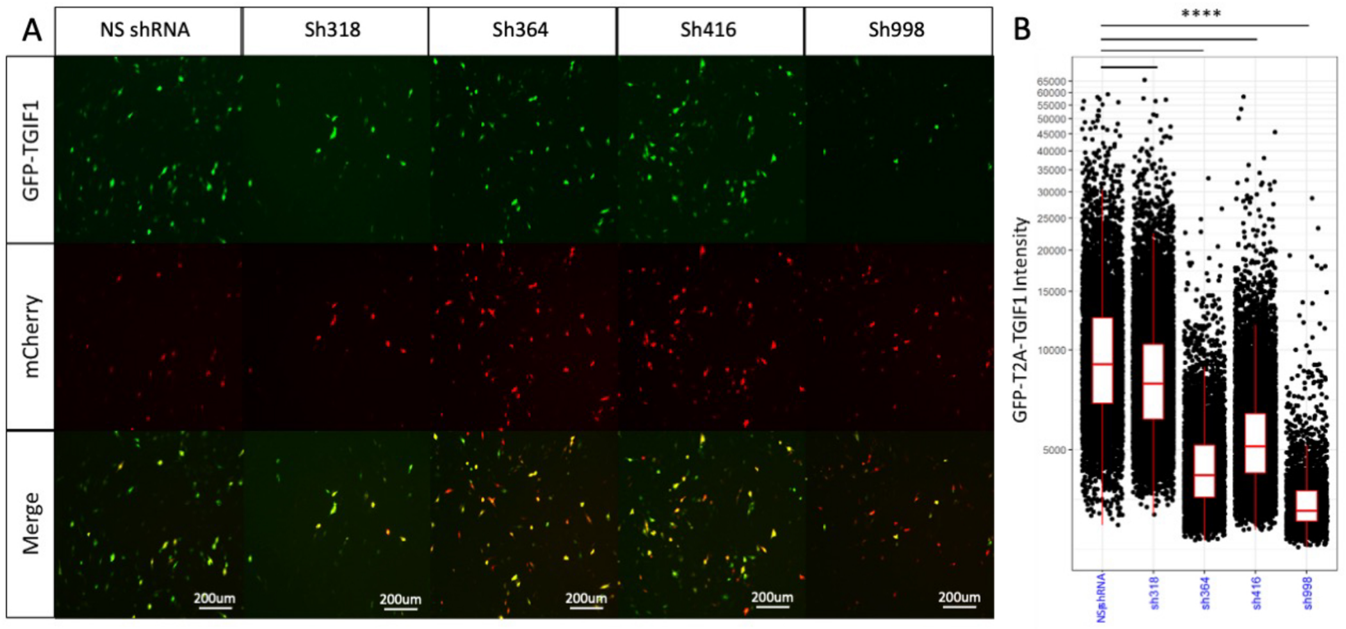

Fig. S6. **TGIF1 sh998 showed the higher repression of TGIF1 in vitro**. DF-1 cells were transfected with a self-replicative viral plasmid containing BFP-T2A and a non-specific shRNA (NS shRNA) or with 4 different putative shRNA designed for TGIF1 knockdown (sh318, sh364, sh416 or sh998). After all cells were BFP positive, they were transfected with the overexpression TOL2-GFP-T2A-TGIF1 plasmid and a plasmid expressing mCherry (as a transfection control). After 48 hours cells were fixed and analyzed under the microscope. (A) Representative fluorescence images of the outcomes. (B) Imaris analysis of the GFP-T2A-TGIF intensity in mCherry positive (transfected) cells. Box plots show each sample's median, interquartile ranges (IQR) and the whiskers extend to the highest/lowest value within 1.5 x IQR. Each dot represents an individual cell. t-test was performed using NS shRNA as a control condition. Dunnett's multiple comparisons test was used as posttest. **** = $p < 0.0001$.

Development • Supplementary information

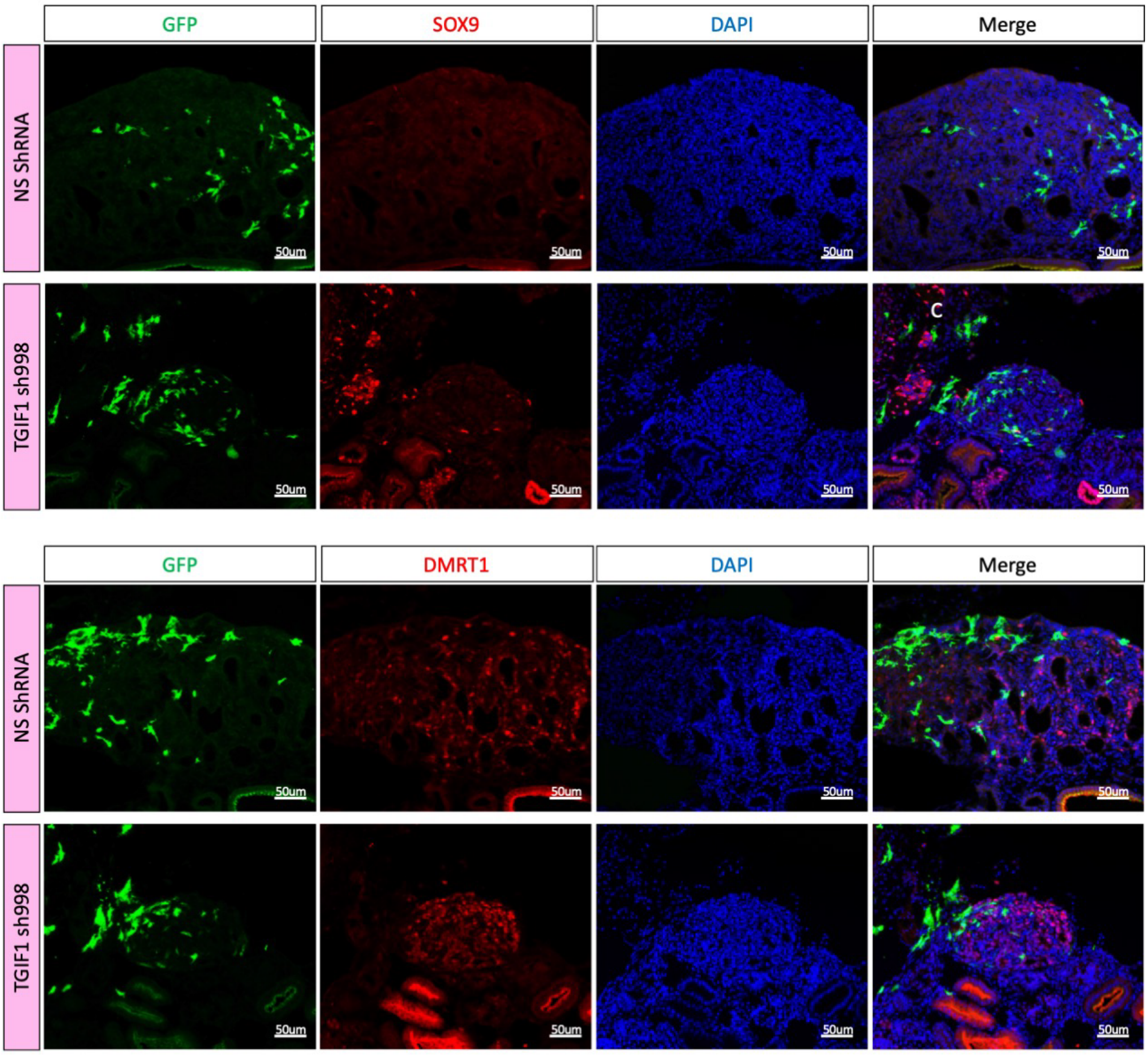

Fig. S7. TGIF1 knockdown does not induce Sertoli cell markers expression. TOL2 TGIF1 knockdown (TGIF1 sh998) or non-silencing control (NS shRNA) plasmids were co-electroporated with a GFP-expressing plasmid (reporter) in female left E2.5 coelomic epithelium. Gonads were examined at E8.5. Immunofluorescence against SOX9 and DMRT1 was performed on transverse sections.

Table S1. Primer list.

Gene and technique	Primer sequence
TGIF1 qPCR	Fw: ACACACCTCTCCACACTACAG<br>Rv: GGTTTGGGTCTTTGCCGTCC
TGIF2 qPCR	Fw: CGCAAACTTTTCGGGGCAC<br>Rv: GGCGCGGTCCTGCTTC
β-Actin qPCR	Fw: GCTACAGCTTCACCACCACA<br>Rv: TCTCCTGCTCGAAATCCAGT
TGIF1 WISH	Fw: AGGAACAAAGGCGAAGCGAG<br>Rv: TTTGGGTCTTTGCCGTCCTT
TGIF1 Overexpression	Fw:GAAAATCCCGGCCCCATGAAAAGTAAAAAAGGTGTAGTTGCAATATCAGG<br>Rv: GTCGTCCTTGTAGTCACTTAGGCCATGAGTTTTGCCTGC
TGIF1 Sh318	Fw: GGCCAGTGAATTCGCGTACCTCCTTCTCGCAGGGC<br>Rv:AGCTATGACGAATTCGCAAAAAACCACTAGATCTTTCCTCATTCTCTTGAAATGAGGAAAGATCTAGTGG<br>AAACCCCAGTTGCTCTCGG
TGIF1 Sh364	Fw: GGCCAGTGAATTCGCGTACCTCCTTCTCGCAGGGC<br>Rv:AGCTATGACGAATTCGCAAAAAAGTGGCAACCTACCCAAAGAGTTCTCTTGAAACTCTTTGGGTAGGTTG<br>CCACAAACCCCAGTTGCTCTCGG
TGIF1 Sh416	Fw: GGCCAGTGAATTCGCGTACCTCCTTCTCGCAGGGC<br>Rv:AGCTATGACGAATTCGCAAAAAAGCACCGATACAATGCTTATTCTCTTGAAATAAGCATTGTATCGGTGC<br>AAACCCCAGTTGCTCTCGG
TGIF1 Sh998	Fw: GGCCAGTGAATTCGCGTACCTCCTTCTCGCAGGGC<br>Rv:AGCTATGACGAATTCGCAAAAAAGGTGGATGTTGCACTCAAATCTCTTGAATTTGAGTGCAACATCCACC<br>AAACCCCAGTTGCTCTCGG
