# Supplementary material for: The homeobox gene TGIF1 is required for chicken ovarian cortical development and generation of the juxtacortical medulla
Source: Development. 2021 Aug 23;148(16):dev199646. doi: 10.1242/dev.199646 (PMC8406534; doi:10.1242/dev.199646)
Supplement: Reviewer comments [file develop-148-199646_review_history.pdf]

The homeobox gene, TGIF1, is required for chicken ovarian cortical development and generation of the juxtacortical medulla

Martin Andres Estermann, Claire Elizabeth Hirst, Andrew Thomas Major and Craig Allen Smith
DOI: 10.1242/dev.199646

Editor: Liz Robertson

Review timeline
Original submission: 30 March 2021
Editorial decision: 29 April 2021
First revision received: 20 June 2021
Accepted: 13 July 2021

Original submission

First decision letter

MS ID#: DEVELOP/2021/199646

MS TITLE: TGIF1 is required for chicken ovarian cortical development and generation of the juxtacortical medulla

AUTHORS: Martin Andres Estermann, Claire E Hirst, Andrew T Major, and Craig A Smith

I have now received all the referees' reports on the above manuscript, and have reached a decision. The referees' comments are appended below, or you can access them online: please go to BenchPress and click on the 'Manuscripts with Decisions' queue in the Author Area.

As you will see, the referees express considerable interest in your work, but have some significant criticisms and recommend a revision of your manuscript before we can consider publication. If you are able to revise the manuscript along the lines suggested, which may involve further experiments, I will be happy receive a revised version of the manuscript. Your revised paper will be re-reviewed by one or more of the original referees, and acceptance of your manuscript will depend on your addressing satisfactorily the reviewers' major concerns. Please also note that Development will normally permit only one round of major revision.

We are aware that you may be experiencing disruption to the normal running of your lab that make experimental revisions challenging. If it would be helpful, we encourage you to contact us to discuss your revision in greater detail. Please send us a point-by-point response indicating where you are able to address concerns raised (either experimentally or by changes to the text) and where you will not be able to do so within the normal timeframe of a revision. We will then provide further guidance. Please also note that we are happy to extend revision timeframes as necessary.

Please attend to all of the reviewers' comments and ensure that you clearly highlight all changes made in the revised manuscript. Please avoid using 'Tracked changes' in Word files as these are lost in PDF conversion. I should be grateful if you would also provide a point-by-point response detailing how you have dealt with the points raised by the reviewers in the 'Response to Reviewers' box. If you do not agree with any of their criticisms or suggestions please explain clearly why this is so.

Reviewer 1

© 2021. Published by The Company of Biologists under the terms of the Creative Commons Attribution License (https://creativecommons.org/licenses/by/4.0/).

Advance summary and potential significance to field

Estermann et al., have shown in a beautiful and comprehensive set of experiments that the homeobox transcription factor TGIF1 is differentially expressed between chick male and female gonads during gonadal formation with higher expression in the ovary. TGIF1 expression was evident in the cortex and medulla of the ovary. They then showed that TGIF1 expression is influenced by the estrogen pathway and probably acts downstream of estrogen in the ovary. Using over expression of TGIF1 in male gonads and reduced expression of TGIF1 in the ovary they were able to show that TGIF1 is able to induce cortex formation in male gonads and is required for the formation of the cortex and the underlying juxtacortical medulla region in females. This works describes TGIF1 as an important player in cortex and juxtacortical medulla formation of the chick ovary.

Comments for the author

I really enjoyed reading the manuscript. It is very well written with elaborated and comprehensive introduction, very nice experiments and data analysis as well as interesting discussion. I think the authors have conducted comprehensive set of experiments including expression analysis, KO experiments and over expression experiments in both sexes. The results and data presented here are solid and fully support the authors conclusion and the role of TGIF1 in cortex formation of the ovary. The manuscript is very suitable for publication in Development and require only very minor revision.

Minor comments:
1. When performing the shRNA you used the DF1 cell line to assess efficiency. It will be helpful if you could analyse the degree of reduction in TGIF1 expression in the actual gonads. This can be done either by qPCR or by in situ-hybridization. I am aware that when using shRNA you will not always see a decrease in mRNA and sometime the decrease will be at the protein level and I understand you do not have a TGIF1 antibody. Yet, in many cases a decrease in mRNA is also present and it can add to the data. In the cell line you had a decrease of 66%, so the phenotype you are seeing may be under-representation of the real phenotype. This can be added to the discussion.

2. It will be helpful if you could add a BF image of the smaller left ovary in figure 9 upon the shRNA electroporation.

3. It may be interesting to know if TGIF1 function may be conserved in mammals. Perhaps you can look at the Stevant et al scRNA-Seq data and Zhao and Koopman bulk RNA-Seq data sets and analyse TGIF1 and TGIF2 expression in the gonads and within which cells it is expressed. It will be interesting to know if it also has sex differential expression there.

Typos
4. The Ioannidis et al., 2020 paper appears as a BioRvix while it is now accepted, please amend the reference accordingly.

5. Line 106- should say asymmetry rather than symmetry

6. Line 225- should say: “area (Fig. 5B) and the thickness…. (Fig. 5C)

7. Line 298: Remove the second “first”

8. In line 273 you have “data not shown”. Please check with the journal guidelines whether this is allowed as many journals do not accept “data not shown”

<u>Reviewer 2</u>

Advance summary and potential significance to field

This is an interesting manuscript, with data suggesting that TGIF1 is required and sufficient for the formation of the female cortex and the juxtacortical medulla. Especially, this is the first report to

© 2021. Published by The Company of Biologists under the terms of the Creative Commons Attribution License (https://creativecommons.org/licenses/by/4.0/).

show the regulator of JCM formation. This said, I have several concerns which the author should address before acceptance.

Comments for the author

1) The authors mentioned in line 218-219, “GFP was not detected in supporting cells in males (Fig. 6)”, when the authors electroporated GFP vector in male left gonad. I would like to see merged images of EGFP and AMH (or Sox9, DMRT1) to reinforce author’s explanation in this manuscript too.

2) The authors infected RCAS virus expressing TGIF1 and EGFP in male embryos (Fig. S3) in order to transfect TGIF1 into male supporting cells. However, in Fig. S3 pictures, we could not see EGFP signals in supporting cells (Sox9, AMH, DMRT1+) on TGIF1 OE sections. Rather, EGFP positive cells and supporting cells show complimentary patterns on TGIF1 OE, in contrast to control sections, which clearly shows supporting cells expressing EGFP. I am very interested in what happened, but I have a concern about the author’s aim to evaluate the supporting cell differentiation by TGIF1 cell-autonomous OE.

3) TGIF1 KD experiments seems unconvincing (Fig. 8,9). In Fig. 8, the authors transfected EGFP-T2A-TGIF1 and mCherry expressing vectors simultaneously and separately in shRNA expressing DF-1 cells, and detected EGFP intensities between samples. So, the authors evaluated KD efficiencies by EGFP intensities. Additionally, I would like to see KD efficiencies by qPCR or western blotting of TGIF1 as more quantitative and reliable evaluating tools. In Fig. 9, I would like to see whether TGIF1 was actually knocked-down in female gonads by double detection of TGIF1 mRNA (in situ hybridization) and EGFP proteins (immunostaining).

4) As minor points, there are several mis-spellings and mistakes in manuscript, including something serious. “Fig. 7” does not appear in the manuscript. Maybe, Fig. 6 in line 248 means Fig. 7.
In line 106-107, the authors described “However, symmetry is maintained and becomes very pronounced in females”. Is it asymmetry?
In line 343 and 430, maybe the authors forgot to write “in” and “of”, respectively, and in line 375, the authors should write “IN mouse”.
In line 206, GPF means GFP.
In line 262, sh988 is sh998? It is confusing.
The authors described CAGGS and aCAGS and ACAGS. These are same things or not?

<u>Reviewer 3</u>

Advance summary and potential significance to field

In the paper titled “TGIF1 is required for chicken ovarian cortical development and generation of the juxtocortical medulla” the authors investigate the expression pattern and role of the transcription factor TGIF1 in gonadal differentiation in the chicken embryo. In sum, I found this paper to be very well written and organized. In addition to providing a good overview of the literature on this topic, the authors described a number of well thought out over-expression and knockdown experiments that clearly show the important role of TGIF1 in ovary differentiation. The results of this work provide novel insights regarding the process of cortical and juxtocortical medulla formation in chicken ovaries; an area that that has remained poorly understood in this field. Initially I was hoping that TGIF1 would weave into the left/right gonad asymmetry in the chicken. Further background on this strange phenomenon would make the manuscript more accessible.

Comments for the author

Several issues should be addressed in the manuscript:
1. Did the investigators try over-expressing TGIF1 in the right ovary or right testis? It is interesting that expression is initially not biased toward the left ovary. I assume the bias is downstream of PITX2 but some discussion of this and how it is linked with E2 signaling would help.

© 2021. Published by The Company of Biologists under the terms of the Creative Commons Attribution License (https://creativecommons.org/licenses/by/4.0/).

2. When E2 is added to male eggs, the left gonad developed cortical characteristics of an ovary. Does anything change in the right gonad? It is surprising that aromatase is upregulated in the male gonad after E2 treatment (I assume this is the left male gonad, but what happens in the right gonad?)

3. In Fig. 9, does treatment with shRNA's against TGIF1 lead to much smaller ovaries? Is the aromatase domain reduced?

4. Fig. 10 suggests that ERα is directly inducing the expression of TGIF1, leading to epithelial structure maintenance and juxtacortical medulla formation. Although this could certainly be possible, the authors have not provided enough evidence to rule out an indirect effect of estrogen signaling.

Below I highlight a few minor issues and suggestions:

Ln 83: change "proved" to "provided"

Ln93: please add "cells" after "Sertoli"

Ln312: please change to "After hatching, germ cell development …"

Ln 316: please change "medullar" to "medulla"

Ln 331: please change to: "Secondly, it acts on the surface […]".

Ln 343: please change to: " Moreover, TGIF and ER-α are both expressed in the left […]"

Ln 362: please change to "TGIF1 may be acting […]"

Lns 380-396: I found that this paragraph lacked focus, particularly lns 387-392. The point that the authors are trying to make in this section is not clear to me. Please consider either shortening this paragraph or reorganizing and splitting it into two.

Lns 384: please consider changing to "[…], forming a zone called the juxtocortical medulla."

Ln 385: please change "testis" to "testes".

Fig. 9: arrows are missing

First revision

<u>Author response to reviewers' comments</u>

There were three reviews of this manuscript. We thank the reviewers for the constructive feedback provided, which has improved the manuscript. As any formatting of the text is lost in this response box, we encourage that the reviewers read the attached "Reviewers Response" file in the supplementary information.
We address each reviewer point by point.

<u>**Reviewer 1**</u>
1) When performing the shRNA you used the DF1 cell line to assess efficiency. It will be helpful if you could analyse the degree of reduction in TGIF1 expression in the actual gonads. This can be done either by qPCR or by in situ- hybridization. I am aware that when using shRNA you will not always see a decrease in mRNA and sometime the decrease will be at the protein level and I understand you do not have a TGIF1 antibody. Yet, in many cases a decrease in mRNA is also

© 2021. Published by The Company of Biologists under the terms of the Creative Commons Attribution License (https://creativecommons.org/licenses/by/4.0/).

present and it can add to the data. In the cell line you had a decrease of 66%, so the phenotype you are seeing may be under-representation of the real phenotype. This can be added to the discussion.

Response:
Demonstration of *TGIF1* knockdown down is an important point, but proved challenging to do *in vivo*, because:

1) The *TGIF1* knockdown gonads are very small, and we were unable to obtain sufficient RNA per replicate to perform qRT-PCR.
2) We manipulated *TGIF1* specifically- and only - in the outer cortex of the gonad. It is also expressed in the underlying medulla, making qRT-PCR problematic when we did not knock it down also in the medulla. Any knockdown in the cortex would be obscured by the more widespread expression in the medulla. Especially taking into consideration that the cortical region is very reduced after knockdown (not multilayered, less cells), as shown in figure 7E and figure 8.

However, to show knock down of *TGIF1* with shRNA *in vivo*, we have now performed *in situ* hybridization, as this reviewer suggested (new Fig. 7). While *in situ* hybridization is a semi-quantitative approach, it provides both expression level and spatial data. These results are shown in new figure 7. Firstly, in Fig 7A, we demonstrate clear knockdown of of *TGIF1* mRNA when over-expressed *in vitro* (in chicken DF1 cells), using our shRNA-998. Secondly, in Fig. 7B, we detected less mRNA staining in the targeted (left) knockdown gonad in vivo compared to the non-silencing control. Also, we provide data on gonadal area- significantly reduced in the left knockdown gonad (Fig. 7 C and D). Most importantly, Fig. 7E, we show loss of *TGIF1* mRNA on a cell-by-cell basis after knockdown, as revealed by overlaid GFP expression (which reports the shRNA)
While *in ovo* electroporation is inherently variable and gives mosaic results, we show that the cells targeted with the knock down shRNA (GFP positive) were also *TGIF1* mRNA negative (white), indicating an effective knock down (Red arrows in Fig. 7E). Please also note the patently reduced gonadal cortex in the *TGIF1* knockdown gonads compared to non-silencing control shRNA (high mag merge Fig. 7E).

2) It will be helpful if you could add a BF image of the smaller left ovary in figure 9 upon the shRNA electroporation.
Response:
An excellent suggestion. Instead of including the bright field image in figure 9 (now new figure 8), we include it in the *in vivo* knock down whole mount *in situ* hybridization figures (see Fig. 7B -E). We also performed quantifications of the whole gonadal area, as well as the transverse sections area, showing a significant size reduction (Fig. 7C and D).

3) It may be interesting to know if TGIF1 function may be conserved in mammals. Perhaps you can look at the Stevant et al scRNA-Seq data and Zhao and Koopman bulk RNA-Seq data sets and analyse TGIF1 and TGIF2 expression in the gonads and within which cells it is expressed. It will be interesting to know if it also has sex differential expression there.
Response:
In the Zhao and Koopman bulk RNA-Seq data sets, *Tgif1* expression was significantly sexually dimorphic at E13.5 dpc, but highly expressed in males. Tgif2 did not show any dimorphic expression. This is now mentioned in the text (lines 494-500 of revised text). In Stevant et al. single cell mouse gonadal data, there is some Tgif1 enrichment in the Sertoli cells, although we don't know if those expression levels are significant. We consider this information and the comparative aspect of gonadal development and differentiation between mammals and birds interesting. It would be ideal to study the role of Tgif1 in mammalian/mouse gonadal development but its out of the scope of this manuscript.

4) The Ioannidis et al., 2020 paper appears as a BioRvix while it is now accepted, please amend the reference accordingly.
Response: Reference is now updated.

© 2021. Published by The Company of Biologists under the terms of the Creative Commons Attribution License (https://creativecommons.org/licenses/by/4.0/).

5) Line 106- should say asymmetry rather than symmetry
Response: Corrected in the revised manuscript.

6) Line 225- should say: “area (Fig. 5B) and the thickness…. (Fig. 5C)
Response: Corrected in the revised manuscript.

7) Line 298: Remove the second “first”
Response: Corrected in the revised manuscript.

8) In line 273 you have “data not shown”. Please check with the journal guidelines whether this is allowed as many journals do not accept “data not shown”
Response: We weren’t able to find information in the journal guidelines about the data not shown policy. Instead, we found that it’s not allowed to cite not shown data. We would like to know the editorial opinion regarding this issue, but we are keen to remove the data not shown statements or provide/include those (negative) results in the supplementary figures if required.

Reviewer 2
1) The authors mentioned in line 218-219, “GFP was not detected in supporting cells in males (Fig. 6)”, when the authors electroporated GFP vector in male left gonad. I would like to see merged images of EGFP and AMH (or Sox9, DMRT1) to reinforce author’s explanation in this manuscript too.
Response:
Figure 6 is now up-dated (now Fig. 5 in the updated manuscript). It now includes the merge containing DAPI, AMH and GFP. Note that this has also been extended to the other figures.

2) The authors infected RCAS virus expressing TGIF1 and EGFP in male embryos (Fig. S3) in order to transfect TGIF1 into male supporting cells. However, in Fig. S3 pictures, we could not see EGFP signals in supporting cells (Sox9, AMH, DMRT1+) on TGIF1 OE sections. Rather, EGFP positive cells and supporting cells show complimentary patterns on TGIF1 OE, in contrast to control sections, which clearly shows supporting cells expressing EGFP. I am very interested in what happened, but I have a concern about the author’s aim to evaluate the supporting cell differentiation by TGIF1 cell-autonomous OE.
Response:
High magnification views are now shown in Fig. S3 to show that both supporting and non-supporting cells of the gonadal medulla are targeted to RCAS virus electroporation (white arrows show supporting cell co-localization of GFP and Sertoli markers) Note that electroporation - of either TOL2 plasmid or RCAS virus - only targets the surface (coelomic) epithelium. We recently showed that, in chicken, the electroporated surface coelomic epithelial cells can proliferate to give rise to non-supporting cells of the medulla (Estermann et al., *Cell Reports*, 2020). However, when we electroporate RCAS virus, it can then spread by self-propagation from those non-supporting to the neighbouring supporting cells. Hence, in this manuscript, we show GFP (and therefore *TGIF1* delivery) to both supporting and non-supporting cells, as shown now in S3. We now included high magnification views of gonadal regions showing GFP (control or TGIF1) co-localizing with the supporting cell markers (white arrows), and also in non-supporting cells. In addition, If TGIF1 had an effect in down-regulating the Sertoli markers, then no colocalization between GFP and DMRT1/SOX9/AMH should be found. We hope this clarify these results.

3) TGIF1 KD experiments seems unconvincing (Fig. 8,9). In Fig. 8, the authors transfected EGFP-T2A-TGIF1 and mCherry expressing vectors simultaneously and separately in shRNA expressing DF-1 cells, and detected EGFP intensities between samples. So, the authors evaluated KD efficiencies by EGFP intensities. Additionally, I would like to see KD efficiencies by qPCR or western blotting of TGIF1 as more quantitative and reliable evaluating tools. In Fig. 9, I would like to see whether TGIF1 was actually knocked-down in female gonads by double detection of TGIF1 mRNA (in situ

© 2021. Published by The Company of Biologists under the terms of the Creative Commons Attribution License (https://creativecommons.org/licenses/by/4.0/).

hybridization) and EGFP proteins (immunostaining).

Response:

Demonstration of *TGIF1* knockdown is an important point. Unfortunately, we could not perform Western blot due to the lack of a suitable antibody against TGIF1. However, we had alternatives. Firstly, we followed the suggestion and now show robust knockdown of over-expressed *TGIF1* mRNA in DF1 cells *in vitro* in the presence of the specific shRNA. This validates shRNA998. See new Fig 7A. We were unable to conduct qRT-PCR on *TGIF1* knockdown gonads, because we were unable to retrieve sufficient mRNA from these gonads as they are very small, and also because medulla expression of *TGIF1* persists and would swamp out any reduced cortex expression. However, as suggested, we performed *TGIF1 in situ* hybridization of gonads electroporated with *TGIF1* sh998 or non-silencing control, and then conducted GFP immunofluorescence (which reports the shRNA) on sectioned whole mounts. This is now shown in new Figure 7B-E. *TGIF1* mRNA expression was lower in the left targeted gonad following specific knockdown, compared to the non-silencing control (new Fig. 7B). Further, we sectioned these whole mount *in situ* gonads to show where the GFP (and hence shRNA) was delivered (new Fig. 7E). While electroporation is always inherently variable, resulting in mosaic delivery, it can be seen that the cortical cells targeted with the knock down shRNA (GFP positive) were *TGIF1* mRNA negative (white). This indicates an effective knock down (Red arrows in Fig. 7E). Please also note in this new figure the patently reduced gonadal cortex in the *TGIF1* knockdown gonads compared to non-silencing control shRNA (high mag merge Fig 7E).

4) As minor points, there are several mis-spellings and mistakes in manuscript, including something serious. “Fig. 7” does not appear in the manuscript. Maybe, Fig. 6 in line 248 means Fig. 7. **Response:** Corrected in the revised manuscript.

In line 106-107, the authors described “However, symmetry is maintained and becomes very pronounced in females”. Is it asymmetry?

Response: This is asymmetry. Corrected in the revised manuscript.

In line 343 and 430, maybe the authors forgot to write “in” and “of”, respectively, and in line 375, the authors should write “IN mouse”.

Response: Corrected in the revised manuscript. In line 206, GPF means GFP.

Response: Corrected in the revised manuscript. In line 262, sh988 is sh998? It is confusing.

Response: It is sh998. Corrected in the revised manuscript.

The authors described CAGGS and aCAGS and ACAGS. These are same things or not?

Response: They refer to the same promoter. They were unified to CAGGS in the newer version of the manuscript.

Reviewer 3

1) Did the investigators try over-expressing TGIF1 in the right ovary or right testis? It is interesting that expression is initially not biased toward the left ovary. I assume the bias is downstream of PITX2 but some discussion of this and how it is linked with E2 signaling would help.

Response:

This is an interesting point, so we have now delivered *TGIF1* over-expression the right gonad rather than the left. We over-expressed of *TGIF1* in the right coelomic epithelium in both female (Fig. 6) and males (Fig. S4). We found that despite not forming a cortex, the epithelium of the right gonad over-expressing *TGIF1* was thicker suggesting that the epithelial flattening was

© 2021. Published by The Company of Biologists under the terms of the Creative Commons Attribution License (https://creativecommons.org/licenses/by/4.0/).

inhibited. In addition, we also found GFP positive cells accumulating in the JCM area in both sexes, when TGIF1 was over-expressed. This data agrees with our previous overexpression data in the left gonad. Left- Right gonadal asymmetry in the female chicken is mediated by PITX2, which is only expressed in the left gonad and can rescue regression of the right gonad (Guioli and Lovell-Badge, 2007). We expect that PITX2 acts earlier than TGIF1, as the expression of TGIF1 is up-regulated after sex differentiation (E6.5, stage 30), whereas PITX2 is expressed earlier in development (stage 17-27) (Rodríguez-León, 2008). Elegant work reported by Ishimaru et al. in 2008 showed that early Pitx2 expression in the chicken embryonic left gonad blocked local retinoic acid signalling, and that this allows estrogen receptor expression in the left gonadal cortex, which is then required for proper cortical development. We hypothesise that TGIF1 is downstream of both Pitx2 and estrogen signalling, given that TGIF1 expression responds to estrogen modulation (Fig 9). This is now discussed in the manuscript (lines 425-442).

2) When E2 is added to male eggs, the left gonad developed cortical characteristics of an ovary. Does anything change in the right gonad? It is surprising that aromatase is upregulated in the male gonad after E2 treatment (I assume this is the left male gonad, but what happens in the right gonad?).

Response:
It is previously documented, by us and others, that application of E2 to male avian embryos induces left cortical development and gonadal feminisation. See our earlier paper: Bannister et al.2011. Manipulation of Estrogen Synthesis Alters miR202 Expression in Embryonic Chicken Gonads . *Biology of Reproduction* (Fig 2B and qPCR 3A).
From our results, we saw that aromatase was upregulated in the right male gonads treated with E2, as well as TGIF1 mRNA expression.

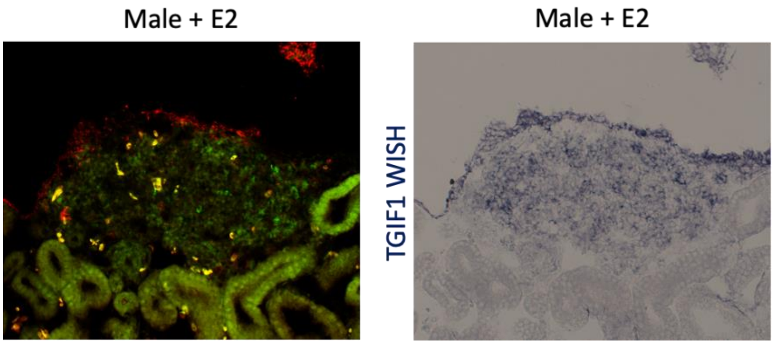

3) In Fig. 9, does treatment with shRNA's against TGIF1 lead to much smaller ovaries? Is the aromatase domain reduced?

Response:
Yes, indeed, TGIF sh998 treatment produced smaller ovaries, which we quantified in the updated version of the manuscript (please see Fig. 7C and D, and compared the control and knockdown gonads in new Figure 7E). As can be seen in figures 7 and 8, the whole gonad was reduced in size, although the medullar/aromatase compartment occupies most of the gonad (Fig 8A).

4) Fig. 10 suggests that ER□ is directly inducing the expression of TGIF1, leading to epithelial structure maintenance and juxtacortical medulla formation. Although this could certainly be possible, the authors have not provided enough evidence to rule out an indirect effect of estrogen signalling.

Response:
True, the response of TGIF1 to estrogen signalling could be indirect. We have updated that figure (now figure 9). Now the arrow has a question mark to evidence the possibility of a direct or indirect effect. This is also highlighted in the text

Ln 83: change “proved” to “provided”
Response: Corrected in the revised manuscript.

© 2021. Published by The Company of Biologists under the terms of the Creative Commons Attribution License (https://creativecommons.org/licenses/by/4.0/).

Ln93: please add “cells” after “Sertoli”
Response: Corrected in the revised manuscript.

Ln312: please change to “After hatching, germ cell development …”
Response: Corrected in the revised manuscript.

Ln 316: please change “medullar” to “medulla”
Response: Corrected in the revised manuscript.

Ln 331: please change to: “Secondly, it acts on the surface […]”.
Response: Corrected in the revised manuscript.

Ln 343: please change to: “ Moreover, TGIF and ER-α are both expressed in the left […]”
Response: Corrected in the revised manuscript.

Ln 362: please change to “TGIF1 may be acting […]”
Response: Corrected in the revised manuscript.

Lns 380-396: I found that this paragraph lacked focus, particularly lns 387-392. The point that the authors are trying to make in this section is not clear to me. Please consider either shortening this paragraph or reorganizing and splitting it into two.
Response: We reorganized and edited the information to make the focus of the paragraph clear (lines 455-472).

Lns 384: please consider changing to “[…], forming a zone called the juxtacortical medulla.”
Response: Corrected in the revised manuscript.

Ln 385: please change “testis” to “testes”.
Response: Corrected in the revised manuscript.

Fig. 9: arrows are missing
Response: We believe the figure has now all the required arrows.

Second decision letter

MS ID#: DEVELOP/2021/199646

MS TITLE: The homeobox gene, TGIF1, is required for chicken ovarian cortical development and generation of the juxtacortical medulla.

AUTHORS: Martin Andres Estermann, Claire E Hirst, Andrew T Major, and Craig A Smith
ARTICLE TYPE: Research Article

I am happy to tell you that your manuscript has been accepted for publication in Development, pending our standard ethics checks.

Reviewer 1

Advance summary and potential significance to field

© 2021. Published by The Company of Biologists under the terms of the Creative Commons Attribution License (https://creativecommons.org/licenses/by/4.0/).

The authors have very nicely addressed all my concerns and comments and I recommend to accept the paper for publication.
Congratulations to all the authors.

Comments for the author

None

Reviewer 2

Advance summary and potential significance to field

This is an interesting manuscript, with data suggesting that TGIF1 is required and sufficient for the formation of the female cortex and the juxtacortical medulla. Especially, this is the first report to show the regulator of JCM formation.

Comments for the author

1)The authors mentioned in line 218-219, “GFP was not detected in supporting cells in males (Fig. 6)”, when the authors electroporated GFP vector in male left gonad. I would like to see merged images of EGFP and AMH (or Sox9, DMRT1) to reinforce author’s explanation in this manuscript too.

Response:
Figure 6 is now up-dated (now Fig. 5 in the updated manuscript). It now includes the merge containing DAPI AMH and GFP. Note that this has also been extended to the other figures. Authors show merged images of EGFP and supporting cell markers in Fig.5 and Fig.6. Those pictures are very convincing to reinforce author’s explanation.

2) The authors infected RCAS virus expressing TGIF1 and EGFP in male embryos (Fig. S3) in order to transfect TGIF1 into male supporting cells. However, in Fig. S3 pictures, we could not see EGFP signals in supporting cells (Sox9, AMH, DMRT1+) on TGIF1 OE sections. Rather, EGFP positive cells and supporting cells show complimentary patterns on TGIF1 OE, in contrast to control sections, which clearly shows supporting cells expressing EGFP. I am very interested in what happened, but I have a concern about the author’s aim to evaluate the supporting cell differentiation by TGIF1 cell-autonomous OE.

Response:
High magnification views are now shown in Fig. S3 to show that both supporting and non-supporting cells of the gonadal medulla are targeted to RCAS virus electroporation (white arrows show supporting cell co-localization of GFP and Sertoli markers) Note that electroporation – of either TOL2 plasmid or RCAS virus – only targets the surface (coelomic) epithelium. We recently showed that, in chicken, the electroporated surface coelomic epithelial cells can proliferate to give rise to non-supporting cells of the medulla (Estermann et al., Cell Reports, 2020). However, when we electroporate RCAS virus, it can then spread by self-propagation from those non-supporting to the neighbouring supporting cells. Hence, in this manuscript, we show GFP (and therefore TGIF1 delivery) to both supporting and non-supporting cells, as shown now in S3. We now included high magnification views of gonadal regions showing GFP (control or TGIF1) co-localizing with the supporting cell markers (white arrows), and also in non-supporting cells. In addition, If TGIF1 had an effect in down-regulating the Sertoli markers, then no colocalization between GFP and DMRT1/SOX9/AMH should be found. We hope this clarify these results.

- I recognize EGFP signals in supporting cells in new pictures with higher magnification of Sup Fig.3. I think virus infection works well.

3) TGIF1 KD experiments seems unconvincing (Fig. 8,9). In Fig. 8, the authors transfected EGFP-T2A-TGIF1 and mCherry expressing vectors simultaneously and separately in shRNA expressing DF-1 cells, and detected EGFP intensities between samples. So, the authors evaluated KD efficiencies by EGFP intensities. Additionally, I would like to see KD efficiencies by qPCR or western blotting of TGIF1 as more quantitative and reliable evaluating tools. In Fig. 9, I would like to see

© 2021. Published by The Company of Biologists under the terms of the Creative Commons Attribution License (https://creativecommons.org/licenses/by/4.0/).

whether TGIF1 was actually knocked-down in female gonads by double detection of TGIF1 mRNA (in situ hybridization) and EGFP proteins (immunostaining).

Response:
Demonstration of TGIF1 knockdown is an important point. Unfortunately, we could not perform Western blot due to the lack of a suitable antibody against TGIF1. However, we had alternatives. Firstly, we followed the suggestion and now show robust knockdown of over-expressed TGIF1 mRNA in DF1 cells in vitro in the presence of the specific shRNA. This validates shRNA998. See new Fig 7A. We were unable to conduct qRT-PCR on TGIF1 knockdown gonads, because we were unable to retrieve sufficient mRNA from these gonads as they are very small, and also because medulla expression of TGIF1 persists and would swamp out any reduced cortex expression. However, as suggested, we performed TGIF1 in situ hybridization of gonads electroporated with TGIF1 sh998 or non-silencing control, and then conducted GFP immunofluorescence (which reports the shRNA) on sectioned whole mounts. This is now shown in new Figure 7B-E. TGIF1 mRNA expression was lower in the left targeted gonad following specific knockdown, compared to the non-silencing control (new Fig. 7B). Further, we sectioned these whole mount in situ gonads to show where the GFP (and hence shRNA) was delivered (new Fig. 7E). While electroporation is always inherently variable, resulting in mosaic delivery, it can be seen that the cortical cells targeted with the knock down shRNA (GFP positive) were TGIF1 mRNA negative (white). This indicates an effective knock down (Red arrows in Fig. 7E). Please also note in this new figure the patently reduced gonadal cortex in the TGIF1 knockdown gonads compared to non-silencing control shRNA (high mag merge Fig 7E).

- Authors quantitatively examined the amount of TGIF mRNA by qPCR in Fig.7 for revision. They show that the amount of TGIF transcripts greatly reduced when TGIF mRNA was knocked down. They demonstrate that their knock-down system is effective sufficiently.

4) As minor points, there are several mis-spellings and mistakes in manuscript, including something serious. “Fig. 7” does not appear in the manuscript. Maybe, Fig. 6 in line 248 means Fig. 7.

Response: Corrected in the revised manuscript.
- Authors handled all of them.

Authors have clarified all my queries. I think this manuscript is acceptable for Development.

<u>Reviewer 3</u>

Advance summary and potential significance to field

In this manuscript, the authors report a role for TGIF2 in the elaboration of the ovarian cortex and formation of the juxtacortical medulla. They show this gene acts downstream of E2 signaling. Over-expression of TGIF2 is not sufficient to form an ovarian cortex in ZZ animals, or in the right ovary of ZW females, thus other genes downstream of E2 are likely involved.

Comments for the author

The authors have made the suggested corrections wherever possible and have improved the manuscript, including interesting OE experiments to address effects on the right gonad in females and males. The evidence that the shRNA effectively reduces TGIF expression in the cells infected by the virus (Fig. 7) is greatly improved. The discussion extends to 6 pages and is very repetitive. I believe it would improve the impact of the work to condense this discussion to the most salient points.

© 2021. Published by The Company of Biologists under the terms of the Creative Commons Attribution License (https://creativecommons.org/licenses/by/4.0/).
